# The novel roles of RNA m^6^A modification in regulating the development, infection, and oxidative DNA damage repair of *Phytophthora sojae*

**DOI:** 10.1371/journal.ppat.1012553

**Published:** 2024-09-23

**Authors:** Fan Zhang, Borui Zhang, Tongshan Cui, Shanshan Chen, Can Zhang, Zhiwen Wang, Xili Liu

**Affiliations:** 1 Department of Plant Pathology, College of Plant Protection, China Agricultural University, Beijing, China; 2 State Key Laboratory of Crop Stress Biology for Arid Areas, College of Plant Protection, Northwest A&F University, Yangling, China; Utrecht University Faculty of Science: Universiteit Utrecht Faculteit Betawetenschappen, NETHERLANDS, KINGDOM OF THE

## Abstract

*N*^6^-methyladenosine (m^6^A), a vital post-transcriptional regulator, is among the most prevalent RNA modifications in eukaryotes. Nevertheless, the biological functions of m^6^A in oomycetes remain poorly understood. Here, we showed that the *PsMTA1* and *PsMTA2* genes are orthologs of human METTL4, while the *PsMET16* gene is an ortholog of human METTL16. These genes are implicated in m^6^A modification and play a critical role in the production of sporangia and oospores, the release of zoospores, and the virulence of *Phytophthora sojae*. In *P*. *sojae*, m^6^A modifications are predominantly enriched in the coding sequence and the 3’ untranslated region. Notably, the *PsMTA1* knockout mutant exhibited reduced virulence, attributed to impaired tolerance to host defense-generated ROS stress. Mechanistically, PsMTA1-mediated m^6^A modification positively regulates the mRNA lifespan of DNA damage response (DDR) genes in reaction to plant ROS stress during infection. Consequently, the mRNA abundance of the DDR gene *PsRCC1* was reduced in the single m^6^A site mutant ΔRCC1/RCC1^A2961C^, resulting in compromised DNA damage repair and reduced ROS adaptation-associated virulence in *P*. *sojae*. Overall, these results indicate that m^6^A-mediated RNA metabolism is associated with the development and pathogenicity of *P*. *sojae*, underscoring the roles of epigenetic markers in the adaptive flexibility of *Phytophthora* during infection.

## Author summary

In this investigation, we embarked on delineating the m^6^A modification landscape in oomycetes, shedding light on the pivotal involvement of PsMTA1, PsMTA2, and PsMET16 in orchestrating m^6^A modification critical for the developmental processes and infection dynamics of *Phytophthora sojae*. Notably, our study underscores the significant impact of PsMTA1-mediated m^6^A modification on the virulence of *P*. *sojae*, particularly in its regulation of the expression of DNA damage response genes prompted by the ROS stress induced by host defenses during infection. This underscores the role of epigenetic variation as a mechanism facilitating adaptive infection strategies in plant pathogens, thus providing a conceptual framework for the design of innovative oomycete inhibitors rooted in epigenetic regulation. We firmly believe that these pioneering insights hold substantial interest for the broader scientific community.

## Introduction

Epigenetic modifications, including histone modifications and DNA methylation, are pivotal in various biological processes in eukaryotes. Recently, RNA modifications have emerged as a significant area of research, recognized as a crucial component of epi-transcriptomic regulation [1–5]. In the past few decades, over a hundred RNA modifications have been identified in both cellular and viral RNA. However, the role of messenger RNA (mRNA) modifications in epigenetics remains under-explored, primarily due to their low abundance and the technical limitations in detecting these modifications [6–7]. Among post-transcriptional modifications, *N*^6^-methyladenosine (m^6^A) is one of the most prevalent RNA modifications. m^6^A modification is crucial for various molecular and cellular processes, including gene expression, alternative splicing, RNA nuclear export, mRNA stability, and translation efficiency [8–12]. Recently, the significance of m6A in viruses, fungi, plants, humans, and other mammals has been increasingly recognized. Numerous studies have investigated the roles of m^6^A in development [13–16]. However, the biological functions of m^6^A modifications in plant pathogens, particularly in oomycetes, remain largely unknown, and it is unclear whether these modifications play a role in the pathogenic infection.

In mammals, m^6^A is catalyzed by the RNA methyltransferase complex (MTC), which primarily comprises methyltransferase-like 3 (METTL3), METTL14, Wilms tumor 1–associated protein (WTAP), KIAA1429, and RNA binding motif protein 15 (RBM15) [17–21], and zinc finger CCCH domain-containing protein 13 (ZC3H13) [22]. METTL3 functions as the core catalytic subunit of the complex. Although METTL14 lacks catalytic activity, it is believed to play a crucial role in substrate recognition [23,24]. Additionally, METTL16, a homolog of METTL3, has been identified as a dynamic regulator of m^6^A in U6 small nuclear RNA (snRNA) and certain mRNAs, thereby influencing intracellular S-adenosylmethionine (SAM) levels [25,26]. Other methyltransferases, including METTL5, ZCCHC4, and METTL4, have also been recognized for their roles in catalyzing m6A modifications [27–29]. Notably, recent findings indicate that METTL4 functions as an m6A writer, methylating U2 snRNA at internal sites in *Drosophila melanogaster* [29]. As core components, disruptions in METTL3 and METTL14 can lead to disturbances in numerous physiological processes, including hematopoiesis and memory cell development [30,31]. YTH domain proteins typically act as m^6^A readers, regulating processes such as leaf formation and trichome morphology [32]. The loss of the m^6^A-binding protein YTHDF1 has been shown to induce neuronal injury in mammals by inhibiting axonal regeneration [33]. Collectively, an increasing body of research underscores the critical role of m6A in normal development and physiological processes across diverse organisms.

Pathogen-associated molecular patterns (PAMPs) induce an outburst of reactive oxygen species (ROS) via transmembrane immune receptors in plants, serving as antimicrobial agents and secondary messengers to trigger the plant immune response [34]. However, the mechanisms by which pathogens tolerate ROS stress remain unclear. ROS can cause DNA damage, including single-strand and double-strand breaks in severe cases [35]. Typically, ROS-induced DNA damage repair involves base excision repair (BER) pathways and double-strand break (DSB) pathways [36,37]. The molecular pathways by which chromatin remodeling regulates DNA repair have recently been elucidated. For instance, in yeast, DNA damage repair mediated by H2A phosphorylation at Serine 139 (γH2Ax) primarily occurs through the recruitment of DNA repair-related proteins [38–40]. However, the regulatory mechanisms of the DNA damage response in oomycetes remain largely unknown.

Oomycetes, a lineage of eukaryotes within the kingdom Stramenopila, comprise numerous pathogens of plants and animals. Morphologically resembling fungi, they are evolutionarily closer to diatoms and brown algae [41–43]. Among these, *Phytophthora* species are highly destructive phytopathogenic oomycetes responsible for severe agricultural devastation worldwide [42–45]. *Phytophthora sojae* is the pathogen responsible for soybean root and stem rot, resulting in an annual economic loss estimated at approximately $1–2 billion. It has been established as a model organism [43–45]. In addition to the wealth of available genomic and transcriptomic data, the clustered regularly interspaced short palindromic repeats (CRISPR) systems have significantly advanced research on the functional genome of *P*. *sojae* [46–48].

In this study, we discovered that PsMTA1 mediates m^6^A modification of DDR gene transcripts, including PsRCC1, presenting a novel regulatory mechanism for tolerating host defense-generated ROS stress in *P*. *sojae*. Our findings underscore the significance of mRNA m6A modification in the development and pathogenesis of P. sojae, laying a theoretical foundation for the development of new fungicides targeting critical proteins in epigenetic regulatory pathways.

## Results

### Identification of m^6^A methyltransferase in *P*. *sojae*

**Fig 1:**
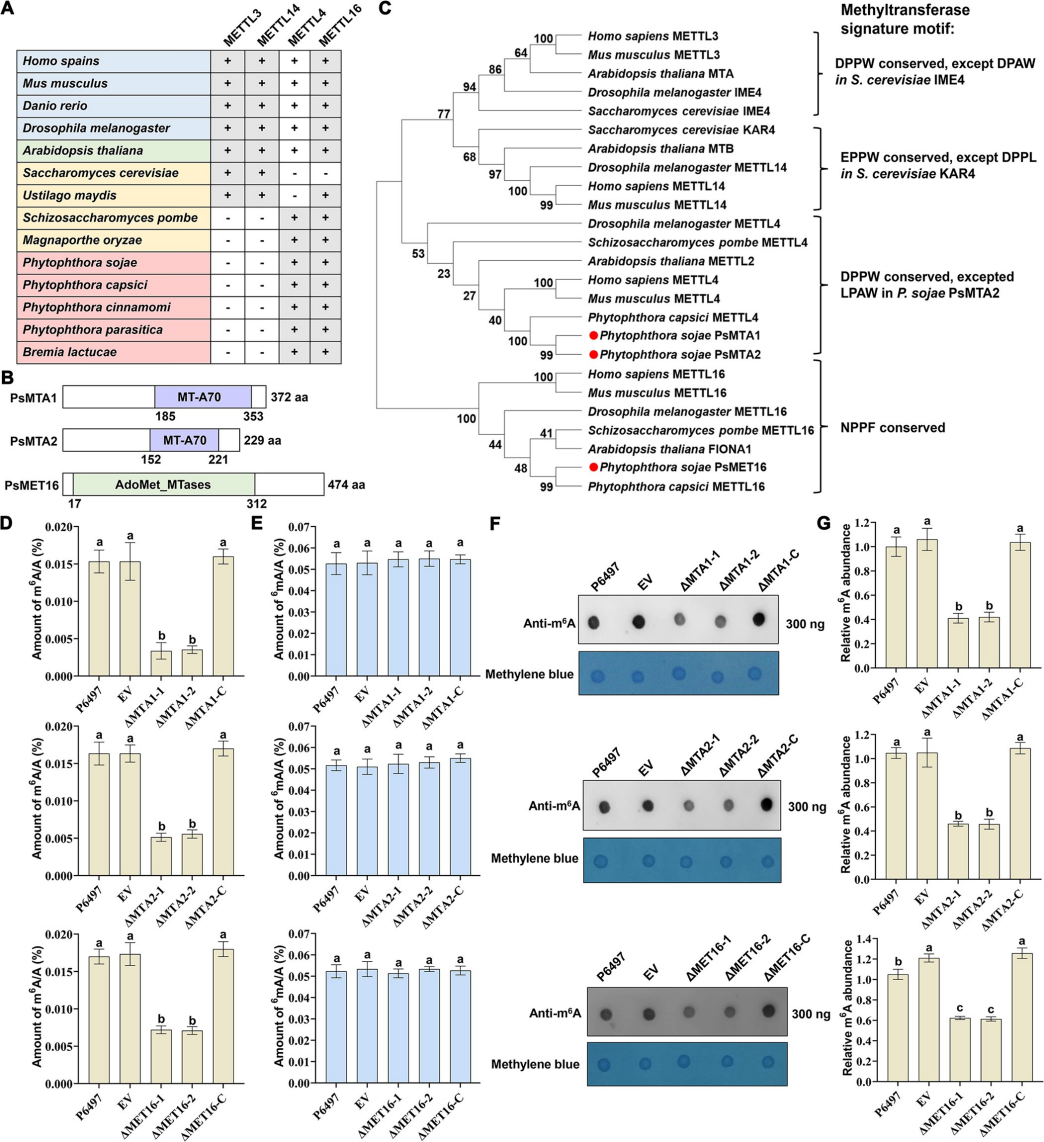
PsMTA1, PsMTA2, and PsMET16 are involved in m^6^A RNA methylation in *Phytophthora sojae*. (**A**) Distribution of human METTL3, METTL14, METTL4, and METTL16 homologue proteins in different organisms. Gray represents the presence of the homologous gene in a species, and white represents the absence of the gene in a species. (**B**) *Phytophthora sojae* MTA1, MTA2, and MET16 proteins conserved domains predicted using a simple modular architecture research tool (Smart). The PsMTA1 and PsMTA2 proteins contain an S-adenosylmethionine-binding domain MT-A70. The PsMET16 protein contains an S-adenosylmethionine-dependent methyltransferases domain Adomet_MTases. (**C**) Phylogenetic analysis of *P*. *sojae* MTA1, MTA2, and MET16 with the homologues and different RNA methyltransferases from other species using molecular evolutionary genetics analysis v.5 (Mega5). Bootstrap analyses were performed with 1000 iterations, and support for each node is displayed. Numbers at the nodes indicate the bootstrap values on neighbor joining analysis. (**D**) Methylation level of m^6^A RNA in the P6497, EV, ΔMTA1-1, ΔMTA1-2, ΔMTA2-1, ΔMTA2-2, ΔMET16-1, ΔMET16-2, ΔMTA1-C, ΔMTA2-C, and ΔMET16-C was detected using an RNA methylation quantitative kit. Different letters represent significant differences by one-way ANOVA (P<0.05). (**E**) Methylation level of ^6^mA DNA in the P6497, EV, ΔMTA1-1, ΔMTA1-2, ΔMTA2-1, ΔMTA2-2, ΔMET16-1, ΔMET16-2, ΔMTA1-C, ΔMTA2-C, and ΔMET16-C was detected using an DNA methylation quantitative kit. Different letters represent significant differences by one-way ANOVA (P<0.05). (**F**) RNA dot blot analysis of m^6^A levels in P6497, EV, ΔMTA1-1, ΔMTA1-2, ΔMTA2-1, ΔMTA2-2, ΔMET16-1, ΔMET16-2, ΔMTA1-C, ΔMTA2-C, and ΔMET16-C using a specific m^6^A antibody. Total RNA from above strains were extracted and spotted onto Hybond™-N+ membranes and incubated with an m^6^A antibody, then detected using the enhanced chemiluminescence (ECL) detection system. Methylene blue staining served as a loading control. (**G**) The relative m^6^A abundance was calculated with the signal density quantified by Image J. Different letters represent significant differences by one-way ANOVA (P<0.05). Data in (**D**, **E**, and **G**) are presented as the mean ± standard deviation from three biological replicates.

To investigate m^6^A modification in *P*. *sojae*, we sought homologs of the *N*^6^-adenosine methyltransferase catalytic subunit METTL3 and the noncatalytic subunit METTL14 [49,50] in the *P*. *sojae* genome database. However, these homologs were not found in oomycetes, including *P*. *sojae* (Fig 1A). Intriguingly, using human METTL4, another N6-adenine-specific methyltransferase, as a reference, we identified two homologous proteins in *P*. *sojae*, designated as MT-A70 domain protein 1 (PsMTA1, Ps382739) and MT-A70 domain protein 2 (PsMTA2, Ps247202), respectively. Additionally, we identified a homolog of human METTL16 in *P*. *sojae*, named PsMET16 (Ps486208). Notably, the genomic sequence of *PsMTA1* was incomplete, necessitating gene correction and annotation (S1A Fig). The corrected *PsMTA1* encodes a protein of 372 amino acids, while *PsMTA2* encodes a protein of 229 amino acids, both containing an MT-A70 domain. PsMET16 comprises 474 amino acids with a typical AdoMet_Mtases domain (Fig 1B). Phylogenetic analysis confirmed that PsMTA1 and PsMTA2 are closer to human and mouse METTL4 but distant from human METTL3, METTL14, or *S*. *cerevisiae* IME4. PsMET16 clusters with human and mouse METT16 on the evolutionary tree (Fig 1C). Examination of the expression patterns of *PsMTA1*, *PsMTA2*, and *PsMET16* throughout all life stages revealed that PsMTA1 and PsMTA2 were up-regulated during asexual reproduction and infestation stages compared to mycelial stages, with *PsMTA2* exhibiting similar expression profiles. PsMET16 displayed up-regulated expression during sporangia and early infection stages (S1B Fig). These findings suggest that PsMTA1, PsMTA2, and PsMET16 may play pivotal roles across various developmental stages and during infection.

To further investigate the putative m^6^A regulatory roles of PsMTA1, PsMTA2, and PsMET16, we generated *PsMTA1*, *PsMTA2*, *and PsMET16* knockout mutants as well as complemented mutants using CRISPR-Cas9 mediated gene strategy, respectively (S1C Fig and S1 Table). The deletion mutants were screened using PCR and confirmed by qRT-PCR S1D and S1E Fig). Six knockout mutants, ΔMTA1-1, ΔMTA1-2, ΔMTA2-1, ΔMTA2-2, ΔMET16-1, and ΔMET16-2, and three complemented knockout mutants, ΔMTA1-C, ΔMTA2-C, and ΔMET16-C, were used for further examination. To determine whether PsMTA1, PsMTA2, and PsMET16 are required for m^6^A RNA methylation or 6mA DNA methylation in *P*. *sojae*, we compared total m^6^A RNA methylation levels between the wild-type strain P6497, empty vector control strain (EV), *PsMTA1*, *PsMTA2*, *and PsMET16* knockout mutants as well as complemented mutants. The amount of m^6^A RNA in the ΔMTA1-1, ΔMTA1-2, ΔMTA2-1, ΔMTA2-2, ΔMET16-1, and ΔMET16-2 mutants showed significant reduction compared to that in P6497, EV, ΔMTA1-C, ΔMTA2-C, and ΔMET16-C (Fig 1D). The amount of 6mA DNA in the ΔMTA1-1, ΔMTA1-2, ΔMTA2-1, ΔMTA2-2, ΔMET16-1, and ΔMET16-2 mutants were comparable with that in the P6497, EV, ΔMTA1-C, ΔMTA2-C, and ΔMET16-C (Fig 1E), suggesting that PsMTA1, PsMTA2, and PsMET16 maybe not be involved in 6mA DNA methylation. We also detected the levels of m^6^A modification using m^6^A mRNA dot blots, which confirmed that the m^6^A methylation level were significantly reduced in the ΔMTA1-1, ΔMTA1-2, ΔMTA2-1, ΔMTA2-2, ΔMET16-1, and ΔMET16-2 mutants compared with P6497, EV, ΔMTA1-C, ΔMTA2-C, and ΔMET16-C strains (Fig 1F and 1G). Therefore, we considered PsMTA1, PsMTA2, and PsMET16 to be involved in m6A methylation of *P*. *sojae*.

### Deletion of *PsMTA1*, *PsMTA2*, or *PsMET16* affects development of *P*. *sojae*

**Fig 2:**
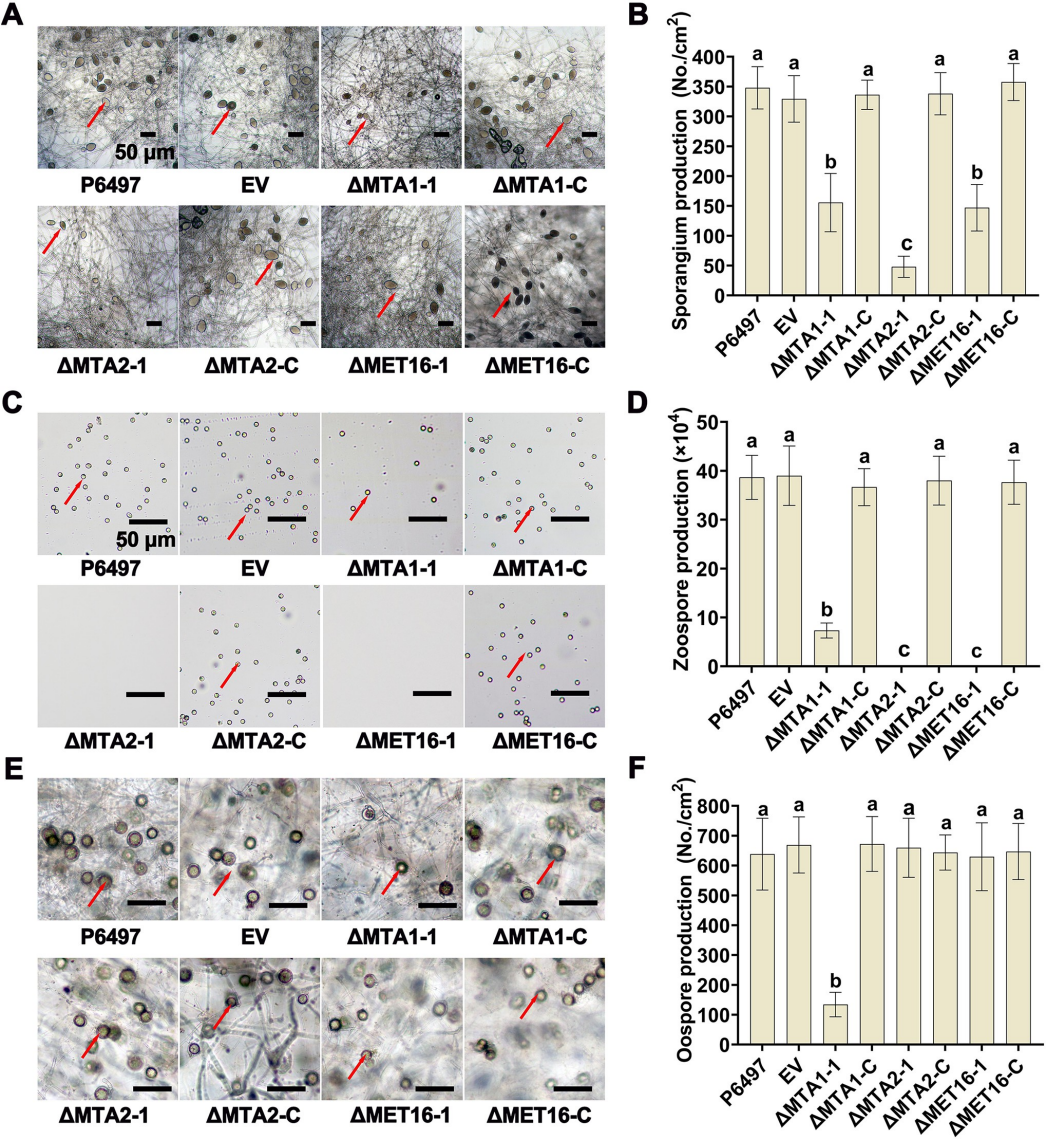
PsMTA1, PsMTA2, and PsMET16 are important for development of *P*. *sojae*. (**A**) Microscopic visualization of sporangia of P6497, EV, ΔMTA1-1, ΔMTA2-1, ΔMET16-1, ΔMTA1-C, ΔMTA2-C, and ΔMET16-C strains. (**B**) Sporangia production. (**C**) Microscopic visualization of zoospores for above strains. (**D**) The number of zoospores released. (**E**) Microscopic visualization of oospores for above strains. (**F**) Oospore production. Scale bar: 50 μm. Different letters represent significant differences by one-way ANOVA (P<0.05). Data in (B, D, and F) are presented as the mean ± standard deviation from three biological replicates.

We then analyzed the phenotypes of *PsMTA1* (ΔMTA1-1), *PsMTA2* (ΔMTA2-1), and *PsMET16* (ΔMET16-1) knockout mutants. When cultured on V8 medium, no significant differences in colony growth diameter were observed in ΔMTA1-1, ΔMTA2-1, and ΔMET16-1 mutants compared to the P6497, EV, ΔMTA1-C, ΔMTA2-C, and ΔMET16-C strains (S2A and S2B Fig). Next, we evaluated the effect of disrupting PsMTA1, PsMTA2, and PsMET16 on stress tolerance in *P*. *sojae*. The results showed that the ΔMTA1-1 and ΔMTA2-1 mutants were more sensitive to a series of stresses, including oxidative stress induced by H_2_O_2_ (10 mM), and osmotic stresses induced by KCl (0.5 M) and sorbitol (0.5 M) ( S2C–S2E Fig). These findings suggest that PsMTA1 and PsMTA2 play important roles in the stress response. In terms of sporangia formation, the P6497, EV, ΔMTA1-C, ΔMTA2-C, and ΔMET16-C strains all produced abundant sporangia. Conversely, the ΔMTA1-1, ΔMTA2-1, and ΔMET16-1 mutants exhibited a significant reduction in sporangia formation (Fig 2A and 2B). We also examined zoospore release in these transformants. The ΔMTA1-1 mutant exhibited a marked decrease in zoospore production compared to P6497, EV, and the complemented strains. Notably, the ΔMTA2-1 and ΔMET16-1 mutants did not release any zoospores (Fig 2C and 2D). Furthermore, the ΔMTA1-1 mutant partially lost its ability to produce oospores, whereas no significant differences were observed in the ΔMTA2-1 and ΔMET16-1 mutants (Fig 2E and 2F). These findings indicate that the deletion of PsMTA1, PsMTA2, or PsMET16 impacts the asexual development of *P*. *sojae*. Additionally, PsMTA1 plays a crucial role in the sexual development of *P*. *sojae*.

### PsMTA1, PsMTA2, and PsMET16 are required for pathogenicity of *P*. *sojae*

**Fig 3:**
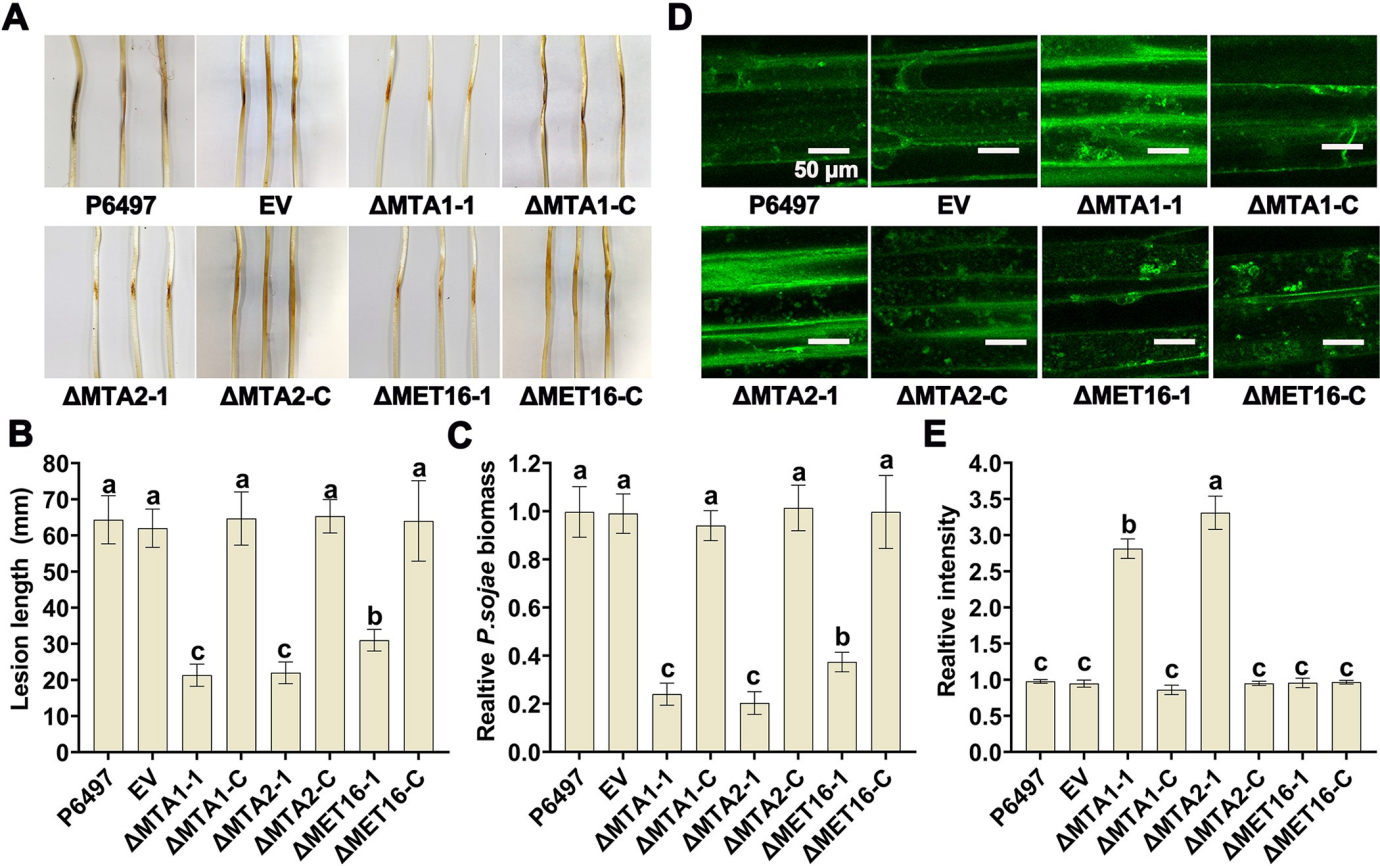
PsMTA1, PsMTA2, and PsMET16 are essential for pathogenicity of *P*. *sojae*. (**A**) Lesions on soybean (cultivar Williams) at 48-hour post inoculation of P6497, EV, ΔMTA1-1, ΔMTA2-1, ΔMET16-1, ΔMTA1-C, ΔMTA2-C, and ΔMET16-C strains. (**B**) Lesion length of above strains. Different letters represent significant differences by one-way ANOVA (P<0.05). (**C**) Relative pathogen biomass in inoculated etiolated hypocotyls expressed as the ratio between the amounts of *P*. *sojae* DNA and soybean DNA detected at 48 hpi with the ratio P6497/soybean set at 1. Different letters represent significant differences by one-way ANOVA (P<0.05). (**D**) ROS production observation in soybean seedlings following above strains infection using the ROS-sensitive fluorescent probe 2’,7’-Dichlorodihydrofluorescein diacetate DCFH-DA. Scale bar: 50 μm. (**E**) The relative relative fluorescence intensity was calculated with the signal density quantified by Image J. P6497 set at 1. Different letters represent significant differences by one-way ANOVA (P<0.05). Data in (**B**, **C**, and **E**) are presented as the mean ± standard deviation from three biological replicates.

To determine whether PsMTA1, PsMTA2, and PsMET16 are involved in the infection process of *P*. *sojae*, we assessed the virulence of P6497, EV, ΔMTA1-1, ΔMTA1-C, ΔMTA2-1, ΔMTA2-C, ΔMET16-1, and ΔMET16-C strains. Soybean hypocotyls inoculated with the ΔMTA1-1, ΔMTA2-1, and ΔMET16-1 mutants developed only small lesions, whereas the P6497, EV, ΔMTA1-C, ΔMTA2-C, and ΔMET16-C strains caused typical disease lesions (Fig 3A and 3B). In infected soybean seedlings, the ΔMTA1-1, ΔMTA2-1, and ΔMET16-1 mutants exhibited reduced pathogen biomass compared to the P6497, EV, ΔMTA1-C, ΔMTA2-C, and ΔMET16-C strains (Fig 3C). To investigate the reduced virulence observed in the ΔMTA1-1, ΔMTA2-1, and ΔMET16-1 mutants, we assessed their ability to activate host immune responses. Pathogen-induced plant immunity is often associated with the accumulation of reactive oxygen species (ROS). We first measured ROS production in soybean seedlings following *P*. *sojae* infection using the ROS-sensitive fluorescent probe 2’,7’-dichlorodihydrofluorescein diacetate (DCFH-DA). As shown in Fig 3D, a substantial accumulation of ROS was observed in soybean seedlings infected with *P*. *sojae* mycelia at 48 hours post-inoculation (hpi). Notably, infection with the ΔMTA1-1 and ΔMTA2-1 mutants resulted in significantly higher ROS levels in host cells compared to infections with the P6497, EV, ΔMTA1-C, ΔMTA2-C, ΔMET16-1, and ΔMET16-C strains (Fig 3D and 3E). When plant epidermal cells were treated with 1 μM of the NADPH oxidase inhibitor diphenyleneiodonium (DPI), which selectively inhibits plasma membrane NADPH oxidase and the generation of extracellular ROS in both plant and mammalian cells, the virulence defect of the ΔMTA1-1 mutants was restored, and the defect in the ΔMTA2-1 mutants was partially compensated S3A and S3B Fig). These findings indicate that PsMTA1, PsMTA2, and PsMET16 play crucial roles in the pathogenicity of *P*. *sojae*, with PsMTA1-mediated m^6^A modification being essential for resistance to ROS stress produced by host defenses.

### MeRIP-seq analysis detects m^6^A modification during infection of *P*. *sojae*

**Fig 4:**
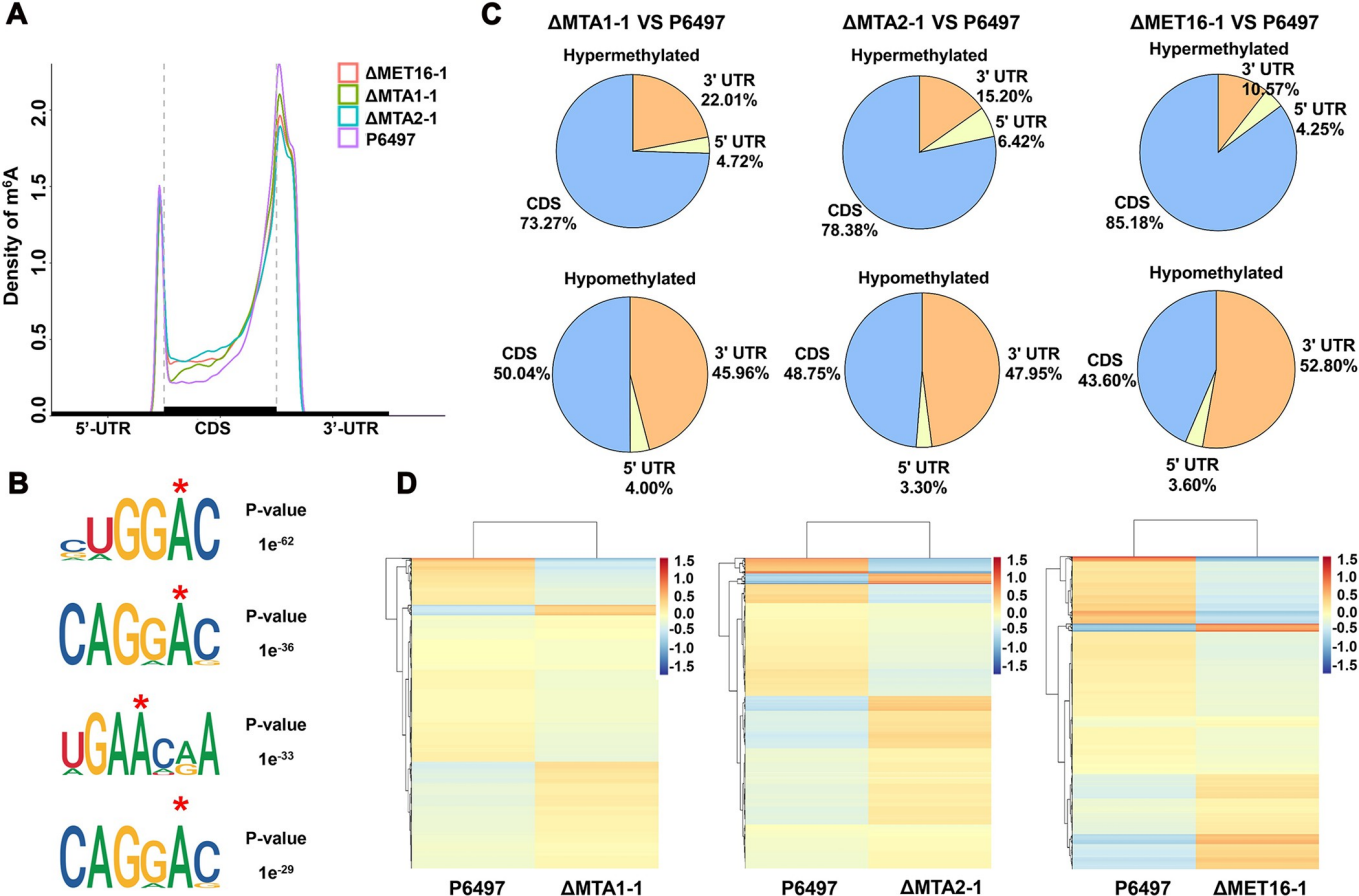
PsMTA1, PsMTA2, and PsMET16-mediated alterations in the m^6^A RNA methylome of *P*. *sojae*. (**A**) Cumulative distribution curve for the level of m^6^A methylation across the wild-type P6497, ΔMTA1-1, ΔMTA2-1, and ΔMET16-1 mutants. (**B**) Consensus sequences identified within m6A peaks of the poly(A) RNAs of *P*. *sojae* from the P6497, ΔMTA1-1, ΔMTA2-1, and ΔMET16-1 mutants. (**C**) Hypermethylated and hypomethylated peaks distribution in the ΔMTA1-1 VS P6497, ΔMTA2-1 VS P6497, and ΔMET16-1 VS P6497. (**D**)Heat map showing m^6^A enrichment values with a statistically significant difference in the ΔMTA1-1 VS P6497, ΔMTA2-1 VS P6497, and ΔMET16-1 VS P6497 (P<0.05).

To elucidate the biological impact of m^6^A modifications in *P*. *sojae* and their underlying mechanisms, particularly in relation to virulence, we conducted m^6^A sequencing on mycelia at 48 hours post-inoculation from strains P6497, ΔMTA1-1, ΔMTA2-1, and ΔMET16-1. The principle underlying this method involves co-immunoprecipitating RNA fragments containing m6A modifications using antibodies specifically designed to recognize these modifications. Subsequent high-throughput sequencing of the precipitated RNA fragments, coupled with comprehensive bioinformatics analysis, allows for a systematic investigation of the m^6^A modification landscape across the entire genome. Among the various techniques employed to study m^6^A modifications, MeRIP-seq (or m^6^A-seq) stands out as one of the most extensively utilized. Approximately 91–98% of clean reads were successfully aligned to the *P*. *sojae* reference genome (JGI v3.0) (S2 Table). Post-processing data analysis allowed for the identification of m^6^A peaks using a peak-calling approach with a P-value threshold of <0.05. Cumulative curve analysis revealed a sequential decrease in the global abundance of m6A modifications in P6497, ΔMTA1-1, ΔMTA2-1, and ΔMET16-1, respectively (S4A Fig). From these data, we identified 12,427, 13,455, 14,456, and 14,326 peaks, respectively, derived from 8,968, 9,221, 9,253, and 9,441 transcripts in the P6497, ΔMTA1-1, ΔMTA2-1, and ΔMET16-1 strains (S4B Fig). We observed a significant enrichment of m^6^A peaks within the 5’ UTR and 3’ UTR regions. Compared to P6497, m6A peaks at the 3’ UTR were notably reduced in the ΔMTA1-1, ΔMTA2-1, and ΔMET16-1 mutants (Figs 4A and S4C). Further analysis using hypergeometric optimization of motif enrichment (HOMER) revealed a motif with the sequence “CUGGAC” as the most frequent putative motif among all the m6A peaks in P6497 (Fig 4B).

To assess the impact of PsMTA1, PsMTA2, and PsMET16 on m^6^A modification, we examined the differential peaks between ΔMTA1-1 and P6497 (ΔMTA1-1 VS P6497), ΔMTA2-1 and P6497 (ΔMTA2-1 VS P6497), and ΔMET16-1 and P6497 (ΔMET16-1 VS P6497). Relative to P6497, ΔMTA1-1 exhibited 353 hypermethylated peaks across 348 genes and 356 hypomethylated peaks across 352 genes. In ΔMTA2-1, there were 628 hypermethylated peaks spanning 598 genes and 426 hypomethylated peaks across 423 genes. Similarly, ΔMET16-1 displayed 477 hypermethylated peaks affecting 453 genes and 411 hypomethylated peaks impacting 407 genes (S4D Fig and S3 Table). Since multiple regions of a transcript may harbor m^6^A modification [51], we scrutinized the number of peaks within each transcript across three distinct comparison groups. The majority of m^6^A-targeted transcripts exhibited a singular m^6^A peak, with only a minority presenting two, three, or more peaks (S4E Fig). Furthermore, Venn diagram results indicated that PsMTA1-, PsMTA2-, and PsMET16-mediated deposition of m^6^A modifications in transcripts partially overlapped (S4F Fig). Relative to P6497, the distribution of hypomethylated peaks in the 3’ untranslated region (UTR) of ΔMTA1-1, ΔMTA2-1, and ΔMET16-1 was notably diminished (Fig 4C). We then examined the characteristics of varying m^6^A levels across different comparison groups. The results revealed significant distinctions among PsMTA1, PsMTA2, and PsMET16 in mediating m^6^A modification in *P*. *sojae* (Fig 4D). To validate the accuracy of the m^6^A-seq data, we randomly selected six m^6^A-modified genes from ΔMTA1-1 VS P6497, ΔMTA2-1 VS P6497, and ΔMET16-1 VS P6497 for validation via MeRIP-qPCR. The alterations in m^6^A levels observed in these six selected transcripts were consistent with the sequencing results, thus confirming the reliability of the sequencing data (S5 Fig and S3 Table).

### Deletion of *PsMTA1*, *PsMTA2*, and *PsMET16* alters the gene expression pattern during infection of *P*. *sojae*

**Fig 5:**
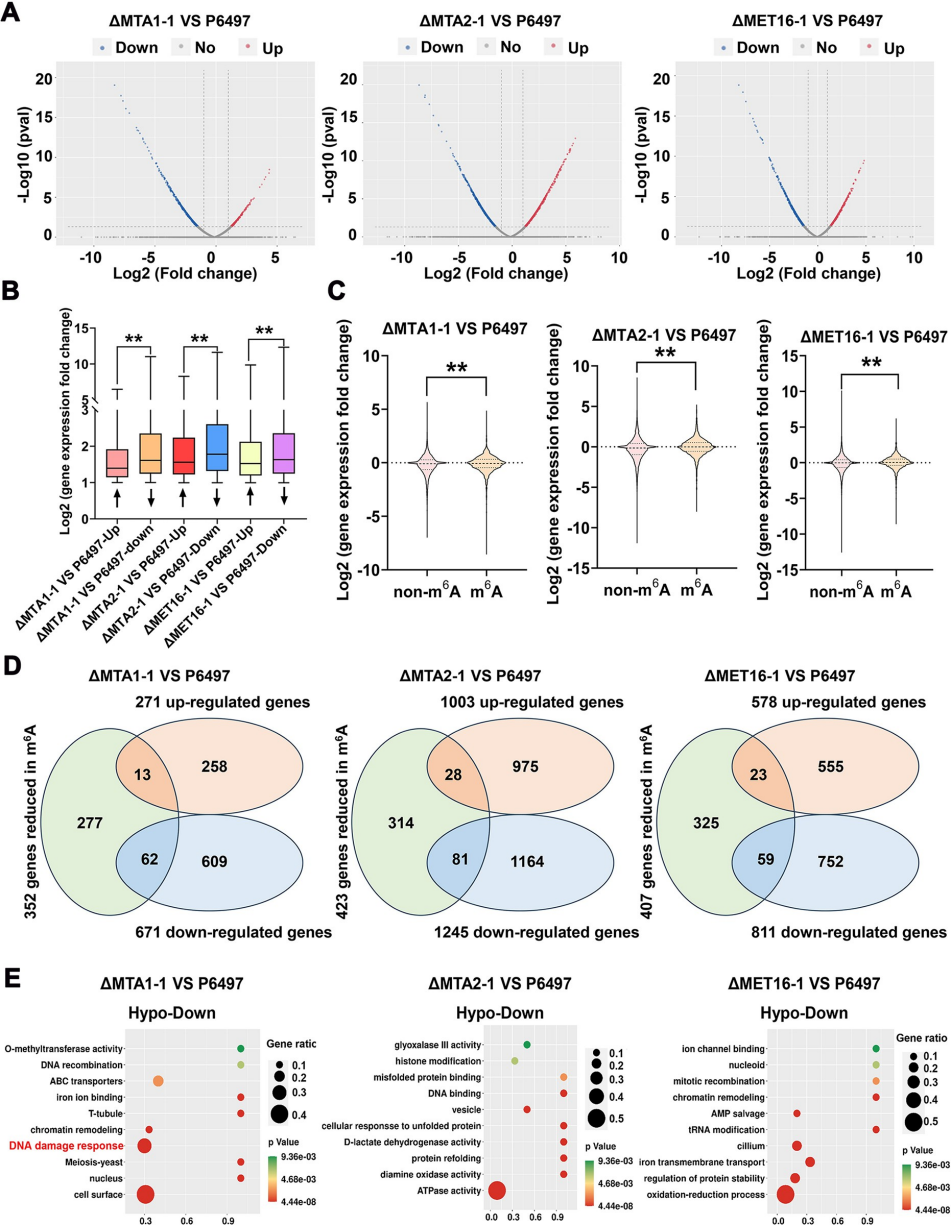
Integrative analysis of m^6^A-seq and RNA-seq identified transcripts affected by PsMTA1, PsMTA2, and PsMET16. (**A**) Volcano plot showing up-regulated genes (red) and down-regulated genes (blue) in the ΔMTA1-1 VS P6497, ΔMTA2-1 VS P6497, and ΔMET16-1 VS P6497. (**B**) Box plots showing the fold change characteristics of up-regulated and down-regulated genes in the ΔMTA1-1 VS P6497, ΔMTA2-1 VS P6497, and ΔMET16-1 VS P6497. (**C**) Boxplot comparison of expression levels of non-m^6^A and m^6^A genes in the ΔMTA1-1 VS P6497, ΔMTA2-1 VS P6497, and ΔMET16-1 VS P6497 (**P < 0.01). (**D**) Venn diagram showing intersection numbers of transcriptionally up-regulated or down-regulated genes and hypomethylated genes in ΔMTA1-1 VS P6497, ΔMTA2-1 VS P6497, and ΔMET16-1 VS P6497. (**E**) Gene Ontology (GO) enrichment of intersection of transcriptionally down-regulated genes with hypomethylated genes in ΔMTA1-1 VS P6497, ΔMTA2-1 VS P6497, and ΔMET16-1 VS P6497.

To comprehend the impact of m^6^A on gene expression at the transcriptional level, we conducted a comparative transcriptome analysis using the same samples harvested from the mycelia at 48 hours post-infection (hpi). Subsequently, we identified 271 up-regulated and 671 down-regulated genes in ΔMTA1-1 VS P6497, 1003 up-regulated and 1245 down-regulated genes in ΔMTA2-1 VS P6497, and 578 up-regulated and 811 down-regulated genes in ΔMET16-1 VS P6497 (fold change > 1.5 and P-value < 0.05) (Figs 5A and S6 and S4 Table). Analysis of the transcriptome data revealed that the number of down-regulated genes and the fold change in expression levels were significantly higher than those of up-regulated genes in all three comparison groups (Fig 5B). Furthermore, comparison of these differentially expressed genes (DEGs) that are not modified by m^6^A with genes subjected to m^6^A modification indicated that the expression levels of m^6^A-mediated DEGs were higher than those of non-m6A DEGs (Fig 5C). These findings suggest that PsMTA1, PsMTA2, and PsMET16-mediated m^6^A modification may regulate gene expression in *P*. *sojae*.

For an integrated analysis aimed at investigating the impact of reduced m^6^A modification on gene expression, we observed that among the 352 hypomethylated mRNAs in ΔMTA1-1 VS P6497, 13 corresponding genes were transcriptionally upregulated, while 62 genes were transcriptionally downregulated. In the case of ΔMTA2-1 VS P6497, among the 423 hypomethylated mRNAs, 28 corresponding genes were transcriptionally upregulated, and 81 genes were transcriptionally downregulated. Similarly, among the 407 hypomethylated mRNAs in ΔMET16-1 VS P6497, 23 corresponding genes were transcriptionally upregulated, and 59 genes were transcriptionally downregulated. These findings suggest that the ΔMTA1-1, ΔMTA2-1, and ΔMET16-1 mutants primarily exhibit a decrease in mRNA abundance (Fig 5D). Gene ontology (GO) enrichment analysis indicated that 62 “hypo-down” genes, which were transcriptionally repressed in the ΔMTA1-1 mutant, were enriched in metabolic pathways related to DNA damage response, nucleus, DNA recombination, and chromatin remodeling-processes closely linked to stress response in *P*. *sojae* (Fig 5E). Additionally, 81 “hypo-down” genes in the ΔMTA2-1 mutant were enriched in pathways associated with ATPase activity, vesicle formation, protein refolding, DNA binding, and diamine oxidase activity. For the ΔMET16-1 mutant, 59 “hypo-down” genes were enriched in pathways involved in oxidation-reduction processes, regulation of protein stability, and iron transmembrane transport (Fig 5E).

### PsMTA1 regulates expression of DNA damage response (DDR) genes during infection of *P*. *sojae*

Given that the loss of pathogenicity in the ΔMTA1-1 mutant is entirely linked to its decreased tolerance to host defense-induced ROS, we further explored the mechanism by which PsMTA1 regulates ROS tolerance during infection. Numerous enzymes are involved in counteracting ROS levels; for instance, catalase mediates the breakdown of H₂O₂. RNA-seq analysis revealed that the transcript levels of catalase homologous genes were not diminished in the ΔMTA1-1 mutant compared to P6497 at 48 hours post-inoculation (hpi) (S7A Fig). In addition, we observed that transcript levels of catalase homologs and catalase activity in ΔMTA1-1, ΔMTA2-1, and ΔMET16-1 mutants were not decreased compared to P6497 (S7B and S7C Fig). These findings indicate that the reduced tolerance to H₂O₂ observed in the ΔMTA1-1 mutant is not due to decreased catalase activity but rather to an impaired ability to repair H₂O₂-induced DNA oxidative damage resulting from the deletion of PsMTA1. Moreover, the ΔMTA1-1 mutant exhibited hypersensitivity to other DNA-damaging agents, such as the alkylating agent mitomycin C (MMC), compared to P6497, EV, ΔMTA1-C, ΔMTA2-1, ΔMTA2-C, ΔMET16-1, and ΔMET16-C (S7D and S7E Fig). This supports the notion that PsMTA1 is primarily involved in DNA damage repair in response to host defense-generated ROS stress.

The integrative analysis of m^6^A-Seq and RNA-seq data indicated that DNA damage response (DDR) transcripts could be crucial targets of PsMTA1 in regulating adaptation to host defense-generated ROS (Fig 5E). Notably, six m6A-modified DDR mRNAs, including *PsMyb* (Myb-like DNA-binding protein; protein ID: Ps327714), *PsCCA1* (circadian rhythm protein; protein ID: Ps286381), *PsGNAT* (GNAT Acetyltransferase family; protein ID: Ps354127), *PsRCC1* (regulator of chromosome condensation; protein ID: Ps487209), *PsBDF1* (Yeast Bromodomain Factor 1; protein ID: Ps325128), and *PsZFAND3* (AN1-type zinc finger protein 3; protein ID: Ps392303), were mediated by PsMTA1 (FDR < 0.05). Their mRNA m^6^A levels were diminished in the ΔMTA1-1 mutant (S8A and S8B Fig and S5 Table). Additionally, we employed strand-specific qRT-PCR to detect the six DDR genes transcripts containing introns, thereby assessing the transcription rate (pre-mRNA) of the six DDR genes. The data showed no significant difference in the transcription rate of the six DDR genes between P6497 and ΔMTA1-1 strain (S8C Fig), suggesting that m^6^A does not influence the initial transcription of the six DDR genes. Subsequent qRT-PCR analysis revealed that these six DDR genes-PsMyb, PsCCA1, PsGNAT, PsRCC1, PsBDF1, and PsZFAND3-were transcriptionally repressed in the ΔMTA1-1 mutant (S8D Fig), suggesting that PsMTA1-mediated m^6^A modification predominantly enhances the expression of DDR genes.

### PsMTA1 mediate the stability of *PsRCC1* mRNA in a m^6^A dependent manner

**Fig 6:**
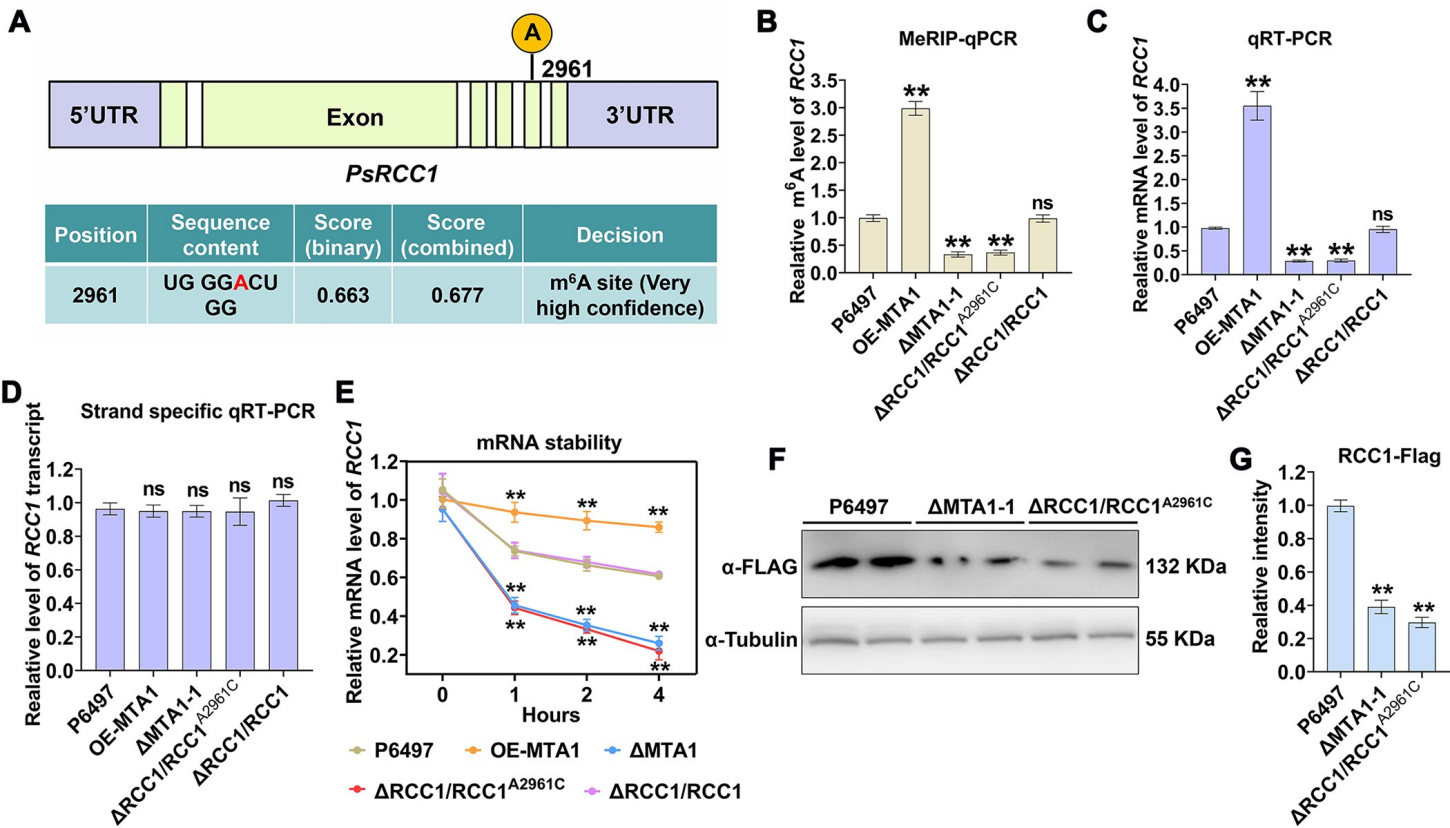
PsMTA1 regulates expression of *P*. *sojae* regulator of chromosome condensation protein 1 (*PsRCC1*) in an m^6^A-dependent manner. (**A**) Online prediction of the m^6^A site of *PsRCC1* based methylation site predictor SNAMP. (**B**) MeRIP-qPCR analysis of m^6^A levels of *PsRCC1* in P6497, OE-MTA1, ΔMTA1-1, ΔRCC1/RCC1^A2961C^, and ΔRCC1/RCC1 mutants. Statistical significance of above strains compared to the P6497 was determined using Student’s t-test (**P < 0.01). (**C**) The mRNA level of PsBdf1 was examined by qRT-PCR in P6497, OE-MTA1, ΔMTA1-1, ΔRCC1/RCC1^A2961C^, and ΔRCC1/RCC1 mutants. Statistical significance of above strains compared to the P6497 was determined using Student’s t-test (**P < 0.01). (**D**) Strand-specific qRT-PCR analysis. Total mRNA was used to perform reverse transcription using specific primer priming the intron region of *PsRCC1* (intron of actin gene was used as a control). qRT-PCR was then performed to amplify the *PsRCC1* intron to demonstrate the transcription rate of *PsRCC1*. Statistical significance of above strains compared to the P6497 was determined using Student’s t-test (ns, no significance). (**E**) The degradation rate of the *PsRCC1* transcripts in the P6497, OE-MTA1, ΔMTA1-1, ΔRCC1/RCC1^A2961C^, and ΔRCC1/RCC1 mutants. The asterisks indicate significant differences compared to 0 h based on Tukey’s test (**P < 0.01). (**F**) Expression of PsRCC1 protein was detected by western blotting with anti-FLAG. Anti-tubulin antibody was used for the control. The asterisks indicate significant differences compared to P6497 based on Tukey’s test (**P < 0.01). (**G**) The intensity of the Flag band from P6497 was set as 1.00; and the relative intensity of PsRCC1-Flag band was quantified with Image J. The asterisks indicate significant differences compared to P6497 based on Tukey’s test (**P < 0.01). Data in (**B**, **C**, **D**, **E** and **G**) are presented as the mean ± standard deviation from three biological replicates.

Previous studies have demonstrated that the regulator of chromosome condensation protein (RCC1) plays a crucial role in the oxidative DNA damage response in both plants and mammals [52,53]. Therefore, *PsRCC1* mRNA might be a critical target of PsMTA1 for the regulation of DNA damage response (DDR). PsRCC1 comprises 1,178 amino acids and features typical Alpha-tubulin suppressor (ATS1) and Protease-associated (PA) domains (S9A Fig). To elucidate this potential mechanism, we first predicted possible m6A sites in PsRCC1 mRNA using the online tool SRAMP (http://www.cuilab.cn/sramp). Notably, a putative m^6^A site at A2961 is located in the CDS region with very high confidence, consistent with the m^6^A-seq results (Fig 6A). To further investigate the regulatory roles of PsRCC1, we generated PsRCC1 knockout mutants (ΔRCC1-1 and ΔRCC1-2) as well as complemented mutants by transforming PsRCC1 into the ΔRCC1-1 mutant (ΔRCC1/RCC1) using a CRISPR-Cas9-mediated gene strategy (S9B and S9C Fig). Additionally, we mutated the A2961 site by substituting A with C and transformed the mutated gene into the ΔRCC1-1 mutant. The resulting transformant, ΔRCC1/RCC1^A2961C^, was used for further analysis. MeRIP-qPCR analysis revealed that, compared with P6497, EV, and ΔRCC1/RCC1, the level of m6A modification on the PsRCC1 mRNA transcript was significantly increased in the overexpression strain OE-MTA1, but noticeably decreased in the ΔMTA1-1 and ΔRCC1/RCC1A^2961C^ mutants (Fig 6B). These results demonstrate that A2961 is a critical m^6^A site on the *PsRCC1* mRNA.

Next, we investigated whether PsMTA1 affects the stability of *PsRCC1* in an m^6^A-dependent manner. qRT-PCR results indicated that the relative expression of PsRCC1 was significantly reduced in the ΔMTA1-1 and ΔRCC1/RCC1^A2961C^ mutants compared to the P6497, EV, and ΔRCC1/RCC1 strains (Fig 6C). Additionally, we employed strand-specific qRT-PCR to detect *PsRCC1* transcripts containing introns, thereby assessing the transcription rate (pre-mRNA) of *PsRCC1*. The data showed no significant difference in the transcription rate of PsRCC1 among P6497, EV, ΔMTA1-1, ΔRCC1/RCC1^A2961C, and ΔRCC1/RCC1 (Fig 6D), suggesting that m^6^A does not influence initial transcription of *PsRCC1*. Furthermore, PsMTA1 markedly enhanced the mRNA stability and protein level of PsRCC1, rather than the translation efficiency of *PsRCC1* mRNA (Figs 6E–6G and S9D). In summary, we confirmed that *PsRCC1* mRNA is a target of PsMTA1, and PsMTA1 mediates the stability of *PsRCC1* mRNA in an m^6^A-dependent manner.

### The m^6^A site A2961 of *PsRCC1* mRNA is important for development and virulence of *P*. *sojae*

Expression patterns of *PsRCC1* throughout all life stages revealed that its expression was upregulated during the asexual reproduction and infestation stages compared to the mycelial stage (S9E Fig). To investigate the impact of the m^6^A site on *PsRCC1* mRNA at position A2961 on the development and virulence of *P*. *sojae*, we assessed the growth rate, asexual and sexual spore production, and virulence of the P6497, EV, ΔRCC1-1, ΔRCC1-2, ΔRCC1/RCC1^A2961C^, and ΔRCC1/RCC1 strains. The results indicated a notable decrease in sporangia and zoospore production in the ΔRCC1-1, ΔRCC1-2, and ΔRCC1/RCC1^A2961C^ strains compared to P6497, EV, and ΔRCC1/RCC1 (S6 Table). More importantly, ΔRCC1-1, ΔRCC1-2, and ΔRCC1/RCC1^A2961C^ caused smaller necrotic lesions compared to P6497, EV, and ΔRCC1/RCC1 (S6 Table). Collectively, the mutation at the A2961 m^6^A site of *PsRCC1* resulted in a significant reduction in the development and pathogenicity of *P*. *sojae*, indicating that this m^6^A site plays a crucial role during infection.

### The m^6^A on *PsRCC1* mRNA A2961 regulates DDR in response to host ROS stress

**Fig 7:**
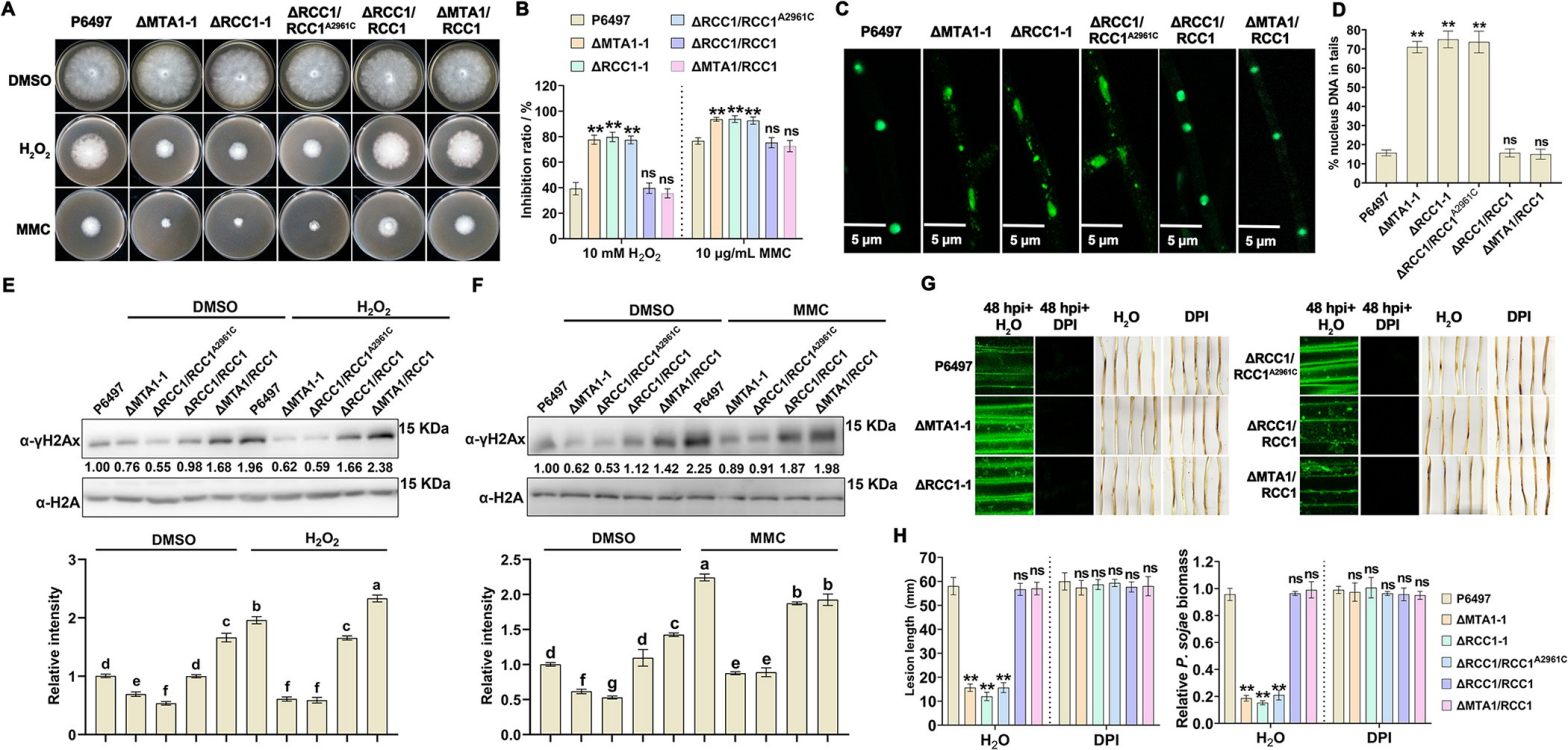
PsMTA1 regulate ROS-induced DNA damage response in an m^6^A-dependent manner. (**A**) Sensitivity detection of P6497, ΔMTA1-1, ΔRCC1-1, ΔRCC1/RCC1^A2961C^, ΔRCC1/RCC1, and ΔMTA1/RCC1 to H_2_O_2_ and alkylating agent mitomycin C (MMC). A 5-mm mycelial plug from the P6497, ΔMTA1-1, ΔRCC1-1, ΔRCC1/RCC1^A2961C^, ΔRCC1/RCC1, and ΔMTA1/RCC1 was incubated on V8 plates supplemented with 0.1% DMSO, 10 mM H_2_O_2_, 10 μg/mL MMC for five days. (**B**) Mycelial growth inhibition the H_2_O_2_ or MMC relative to the DMSO-treated control. Relative inhibition was calculated as (CK−Growth rate on plates with treatment)/CK×100%. Statistical significance of the mycelial inhibition of above strains compared to the wild type P6497 was determined using Student’s t-test (**P < 0.01, ns: not significant). (**C**) Analysis of DNA damage in P6497, ΔMTA1-1, ΔRCC1-1, ΔRCC1/RCC1^A2961C^, ΔRCC1/RCC1, and ΔMTA1/RCC1 by a comet assay following the H_2_O_2_ treatment. The mycelium was cultured in V8 liquid medium for 3d and then used for nuclear staining. (**D**) Quantitative analysis of the DNA in tails of P6497, ΔMTA1-1, ΔRCC1-1, ΔRCC1/RCC1^A2961C^, ΔRCC1/RCC1, and ΔMTA1/RCC1 shown in (**C**). Statistical significance of above strains compared to the P6497 was determined using Student’s t-test (**P < 0.01, ns: not significant). (**E**) The elevation of γH2Ax levels in response to 10mM H_2_O_2_ was inhibited in ΔMTA1-1 and ΔRCC1/RCC1^A2961C^ mutants compared to that in P6497, ΔRCC1/RCC1, and ΔMTA1/RCC1. γH2Ax levels were determined by western blotting using anti-γH2Ax antibody. Detection of H2A protein was used as the loading control (upper panel).The intensity of the γH2Ax band from P6497 treated with DMSO was set as 1.00; and the relative intensity of γH2Ax band from each treatment was quantified with Image J (lower panel). Different letters represent significant differences by one-way ANOVA (P < 0.05). (**F**) The elevation of γH2Ax levels in response to 10 μg/mL MMC was inhibited in ΔMTA1-1 and ΔRCC1/RCC1^A2961C^ mutants compared to that in P6497, ΔRCC1/RCC1, and ΔMTA1/RCC1. γH2Ax levels were determined by western blotting using anti-γH2Ax antibody. Detection of H2A protein was used as the loading control (upper panel).The intensity of the γH2Ax band from P6497 treated with DMSO was set as 1.00; and the relative intensity of γH2Ax band from each treatment was quantified with Image J (lower panel). Different letters represent significant differences by one-way ANOVA (P < 0.05). (**G**) The treatment with 1 μM DPI restored the impaired virulence of ΔMTA1-1, ΔRCC1-1, and ΔRCC1/RCC1^A2961C^. Soybean seedlings were inoculated with mycelial plugs of P6497, ΔMTA1-1, ΔRCC1-1, ΔRCC1/RCC1^A2961C^, ΔRCC1/RCC1, and ΔMTA1/RCC1 and then stained at 48 hpi by DCFH-DA with or without 1 μM DPI as ROS scavenger. Disease symptoms were observed at 48 hpi of above strains when soybean seedlings were treated with H_2_O (control) or DPI. (**H**) Lesion length (left panel) and pathogen biomass (right panel) of each strain under treatments with H_2_O (control) or DPI, measured at 48 hpi. Statistical significance of the lesion length and biomass of above strains compared to the P6497 at H_2_O or DPI treatment was determined using Student’s t-test (**P < 0.01, ns: not significant). Data in (**B**, **D**, **E**, **F**, and **H**) are presented as the mean ± standard deviation from three biological replicates.

Pathogens must tolerate the ROS stress generated by host plants during infection. To investigate whether and how m^6^A-modified PsRCC1 contributes to ROS stress tolerance via mediating the DNA damage response (DDR), we first examined the sensitivity of PsRCC1 mutants to H₂O₂ and MMC. Compared to P6497, EV, and ΔRCC1/RCC1, the ΔRCC1-1 and ΔRCC1/RCC1^A2961C^ mutants exhibited hypersensitivity to both H₂O₂ and MMC (Fig 7A and 7B). To elucidate whether other five DDR related genes (*PsMyb*, *PsCCA1*, *PsGNAT*, *PsBDF1*, and *PsZFAND3*) are genetically involved in ROS response, single knockout mutants of *PsCCA1*, *PsGNAT*, and *PsZFAND3* genes and gene silencing mutants of *PsMyb* and *PsBDF1* were constructed (S10A–S10C Fig). The resulting mutants were tested for sensitivity to H_2_O_2_ on V8 medium. We found that these mutants exhibited no significantly increased sensitivity to H2O2 (S10D Fig). These results suggest that among these DDR genes, PsRCC1 is primarily responsible for the response to ROS-induced DNA damage. To further validate the genetic association between PsMTA1-dependent m^6^A modification and PsRCC1 in mediating the DNA damage response (DDR), we created a PsRCC1 overexpressing strain (ΔMTA1/RCC1) by transforming PsRCC1 into the ΔMTA1-1 mutant (S11 Fig). Interestingly, the sensitivity of ΔMTA1/RCC1 to H₂O₂ and MMC was comparable to that of P6497, EV, and ΔRCC1/RCC1 (Fig 7 and 7B). These results suggest that *PsRCC1* mRNA is a crucial target of PsMTA1-mediated m^6^A modification for the regulation of DDR. To ascertain the roles of m^6^A-modified PsRCC1 in regulating ROS-related DDR, we conducted a comet assay to assess DNA damage in P6497, EV, ΔMTA1-1, ΔRCC1-1, ΔRCC1/RCC1^A2961C^, ΔRCC1/RCC1, and ΔMTA1/RCC1 strains following treatment with 10 mM H_2_O_2_. As anticipated, the proportion of DNA in the tail of ΔMTA1-1, ΔRCC1-1, and ΔRCC1/RCC1^A2961C^ mutants was significantly higher than that in P6497, EV, ΔRCC1/RCC1, and ΔMTA1/RCC1 (Fig 7C and 7D), indicating the involvement of m^6^A-dependent PsRCC1 in ROS-associated DDR. A fundamental mechanism for preserving genome integrity involves the activation and accumulation of γH2Ax at DNA damage sites in an ATM-dependent manner, which recruits repair factors to these sites (Fig 7E). To further evaluate whether PsRCC1 mediates DNA damage repair, we examined the abundance of γH2Ax in the aforementioned strains exposed to H_2_O_2_ and MMC. Significantly, we observed that H_2_O_2_ and MMC treatments induced a notable accumulation of γH2Ax compared to the untreated P6497 strain (DMSO). However, the enrichment of γH2Ax was markedly suppressed in ΔMTA1-1, ΔRCC1-1, and ΔRCC1/RCC1^A2961C^ mutants in comparison to P6497, ΔRCC1/RCC1, and ΔMTA1/RCC1 following H_2_O_2_ and MMC treatments (Figs 7F and S12). Particularly noteworthy is that treatment of plant epidermis cells with DPI at 1 μM resulted in the recovery of virulence defects observed in ΔMTA1-1, ΔRCC1-1, and ΔRCC1/RCC1^A2961C^ mutants (Fig 7G and 7H). In summary, these findings indicate that PsMTA1-regulated PsRCC1 plays a crucial role in DNA damage response and tolerance to ROS stress induced by host defenses in an m^6^A-dependent manner.

## Discussion and conclusions

There is mounting evidence indicating the pivotal role of m^6^A modification in orchestrating RNA metabolism and various biological processes in eukaryotes [13,16]. However, to date, neither the involvement of m^6^A nor the identification of its methyltransferase has been reported in oomycetes. Among the most widely recognized candidates for m^6^A methyltransferases are those belonging to the MT-A70 family, such as human METTL3 and *S*. *cerevisiae* Ime4 [17,18]. In this study, through phylogenetic analysis (Fig 1A), we found that *P*. *sojae* PsMTA1 and PsMTA2 exhibit closer evolutionary proximity to human METTL4, another member of the MT-A70 family, rather than to human METTL3, METTL14, or *S*. *cerevisiae* Ime4. Notably, homologous proteins of METTL4 typically function as DNA N6-methyladenine (6mA) writers across different organisms [54–56]. METTL4 has also been found to regulate m6A methylation of U2 snRNA in Drosophila [29], implying potential roles of this protein in RNA modification. In Drosophila, METTL4 exhibits weak enzymatic activity on DNA substrates but strong enzymatic activity on RNA substrates [29]. Interestingly, our study revealed that the level of DNA 6mA modification did not decrease in ΔMTA1 and ΔMTA2 mutants, suggesting that MTA1 may not be responsible for 6mA in *P*. *sojae*. In contrast, ΔMTA1 and ΔMTA2 mutants exhibited a significant decrease in m^6^A levels, indicating an important role of PsMTA1 and PsMTA2 in the m^6^A modification of *P*. *sojae* (Fig 1F). Furthermore, we identified a human METTL16 homolog, PsMET16, which also regulates global m^6^A modification in *P*. *sojae*. These findings collectively suggest that PsMTA1, PsMTA2, and PsMET16 are essential for efficient m^6^Amodification and gene expression in *P*. *sojae*.

In mammals, m^6^A modifications can influence various RNA-related processes, including splicing, degradation, and translation, with mRNA stability (degradation) being the most extensively studied role [57–59]. In plants, numerous instances of m6A negatively regulating mRNA stability have been documented [60]. For instance, in Arabidopsis, methyltransferases FIP37- and VIRILIZER-induced m^6^A methylation exhibited a negative correlation with transcript stability [14,61,62]. These findings may stem from distinct m^6^A reader-mediated processes occurring in cells or developmental stages across different species. Our findings indicate that m^6^A modification in *P*. *sojae* is more inclined to positively regulate DDR gene expression by influencing mRNA stability. These observations suggest a preference of m^6^A readers in *P*. *sojae* for positively regulating gene expression, although the specific regulatory mechanism warrants further investigation. Furthermore, we noticed the minor changes in the ΔMTA1-1, ΔMTA2-1, and ΔMET16-1 mutants compared to P6497 (Fig 4A). Previous studies have generally indicated that there was no significant difference in the distribution of the m^6^A peaks between the methyltransferase mutant and the parent line. For example, m^6^A modification in the wild-type was significantly enriched within the 5’ UTR and 3’ UTR regions. While, in the Δmta1 mutant, m^6^A peaks at the 3’ UTR were significantly reduced [16]. We hypothesize that the observed increase in methylation peaks in other regions may be attributed to a compensatory mechanism involving other methyltransferase.

The regulatory effect of PsMTA1-, PsMTA2-, and PsMET16-mediated m^6^A modification on the biological phenotype of *P*. *sojae* is mainly achieved by affecting the expression of multiple target genes. Oomycetes and fungi exhibit analogous pathogenic mechanisms. In the context of plant pathogens, prior studies have identified PoIme4 as an N6-adenosine methyltransferase, with the deletion of PoIME4 resulting in reduced levels of m6A RNA methylation. PoYth1 and PoYth2, identified as m^6^A-binding proteins, show functional specificity, as the deletion of PoYTH2 significantly impairs conidiation. Virulence assays further demonstrate that PoIME4, along with PoAlkb1 (an mRNA demethylase), PoYTH1, and PoYTH2, play crucial roles in the pathogenicity of *Pyricularia oryzae* on rice [63]. Moreover, MTA1 has been shown to regulate the m^6^A modification of autophagy-related protein (ATG) gene transcripts, subsequently mediating mRNA degradation. This regulation is critical for maintaining the balance of autophagy within appressoria, facilitating appressorium maturation, and enabling host penetration by the rice blast fungus [16]. Additionally, in *Fusarium graminearum*, MTA1-overexpressing strains (MTA1-OE) exhibit delayed conidial germination and reduced hyphal branching, indicating its involvement in vegetative growth [64]. Our findings parallel these observations, revealing that PsMTA1, PsMTA2, and PsMET16 are integral to the development and virulence of *P*. *sojae*. Notably, while m^6^A often exerts a negative regulatory effect on gene expression in fungi, thereby influencing downstream biological phenotypes [16], our study suggests a more positive regulatory role for m^6^A modification. These seemingly contradictory outcomes may stem from divergent m6A reader-mediated processes across different cellular contexts or developmental stages in various species. Furthermore, while the methyltransferase, demethyltransferase, and reader proteins associated with m^6^A have demonstrated significant biological functions in plant-pathogenic fungi, the corresponding m^6^A demethyltransferase and reader proteins in Phytophthora require further identification and functional characterization.

**Fig 8:**
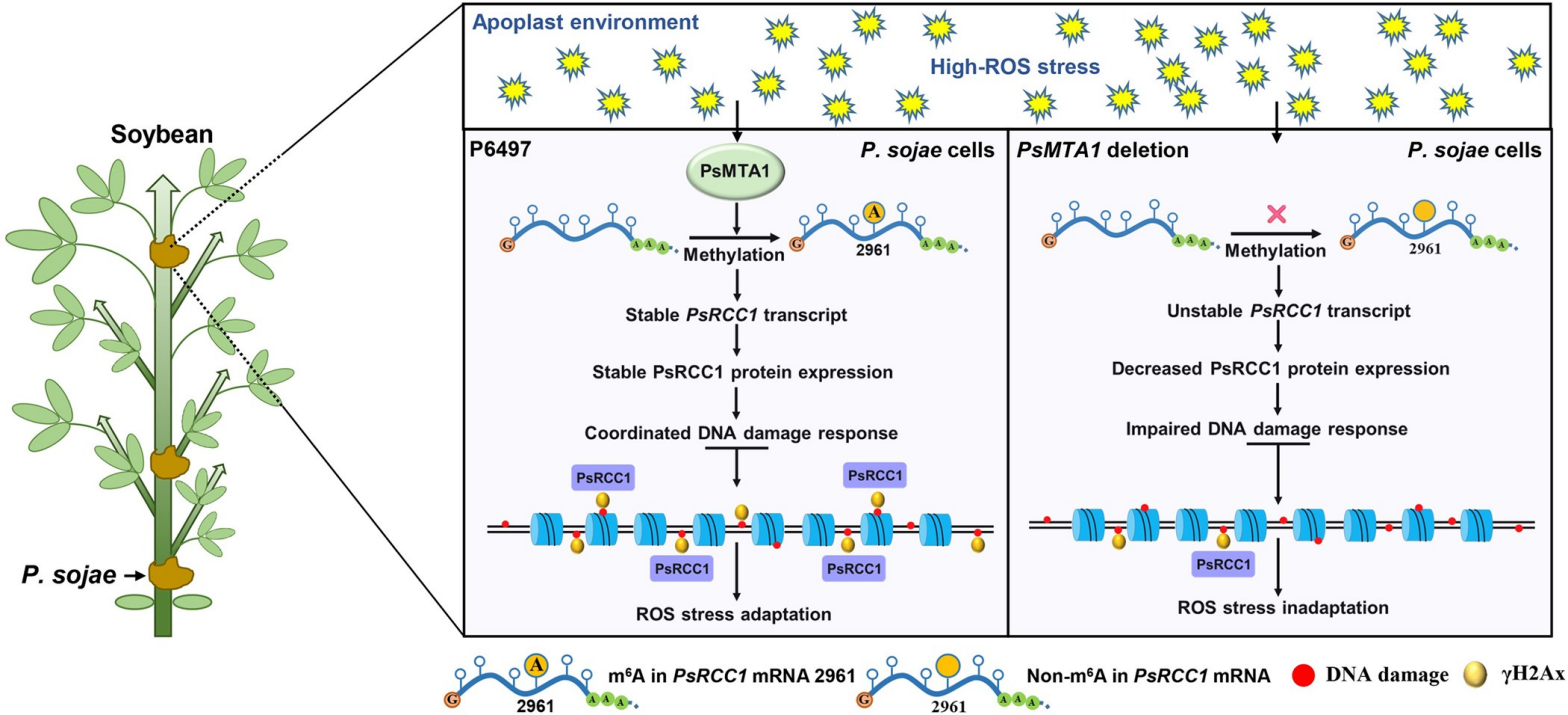
Working model of the mechanism of PsMTA1 regulates ROS-associated DNA damage response in an m^6^A-dependent manner. Our study provides a novel function model of which m^6^A can positively regulate the expression of DDR gene *PsRCC1* to mediate the DNA damage repair induced by host ROS response, and then contribute the pathogen to complete the colonization of host plants. PsMTA1 mediates the m^6^A modification of DDR mRNAs including *PsRCC1*, which can maintain the stability of these DDR gene transcripts. Thus, the *PsRCC1* mRNA level of the DDR genes are well balanced through m^6^A methylation and demethylation, although the de-methylation enzyme requires confirmation. The mRNA level balance contributes to the PsRCC1 protein level balance, in turn, coordinates the DDR process, thereby regulating the tolerance of *P*. *sojae* to plant defense-generated ROS stress during infection.

Recent studies have indicated that the enrichment of m^6^A RNA at UV irradiation sites recruits DNA polymerase κ (POLK) as an early response mechanism for DNA repair [65]. However, the relationship between m^6^A-mediated DNA repair and host invasion by plant pathogens has not been previously explored. In human cells, RCC1 functions as a guanine nucleotide exchange factor (GEF) for Ran GTPase and also exhibits chromatin and DNA binding activity in the nucleus, thereby regulating gene expression [66,67]. In plants, there are 24 genes encoding RCC1 family proteins. Among them, UVR8 and TCF1 (Tolerant to Chilling and Freezing 1) have been extensively studied, with both proteins playing pivotal roles in mediating plant responses to environmental conditions [68–70]. Moreover, RCC1 and ATM cooperatively regulate the alternative splicing of mitochondrial nad2 and the DNA damage response in Arabidopsis thaliana [71]. Intriguingly, our investigation presents a novel functional model wherein m^6^A positively regulates the expression of the DDR gene PsRCC1 to facilitate DNA damage repair induced by host ROS response, thereby promoting the pathogen’s colonization of host plants (Fig 8). PsMTA1 mediates m^6^A modification of DDR mRNAs, including PsRCC1, ensuring the stability of these DDR gene transcripts during infection. Consequently, mRNA levels of DDR genes are finely tuned through m^6^A methylation and demethylation, although the demethylation enzyme necessitates validation. This equilibrium in mRNA levels contributes to a corresponding balance in protein levels, consequently orchestrating the DDR process and regulating *P*. *sojae*’s tolerance to plant defense-induced ROS stress during infection. Our study underscores the functional significance and regulatory mechanism of m^6^A in *P*. *sojae*. These findings offer insights into the underlying molecular mechanisms of m^6^A modification in oomycetes, with potential implications for the development of novel fungicides for pathogen disease management.

## Materials and methods

### *P*. *sojae* assay strains and growth conditions

The wild-type strain P6497, provided by Brett Tyler (Oregon State University, Corvallis, OR, USA), along with all mutants in this study, were cultured on V8 agar or liquid medium at 25°C in the dark. These strains were cultured on V8 plates in the dark at 25°C for 6 days. After this period, 10 mycelial blocks were transferred to a culture dish containing 20 ml of V8 liquid medium, with the mycelial side facing up and partially submerged. The V8 liquid medium was then decanted, and the mycelia were rinsed with 20 ml of sterile water five times at 30-minute intervals. Subsequently, 20 ml of sterile water was added, and the cultures were incubated in the dark at 25°C for 8–12 hours to induce sporangium formation. The number of sporangia produced by each strain was then examined microscopically in each visual field. Three biological replicates were performed. Zoospores were collected following the method described above [45].

### Targeted gene knockout and complementation

Gene knockout mutants were generated using CRISPR-mediated gene editing. We employed PEG/CaCl2-mediated transformation of P. sojae protoplasts [46,47]. Candidate transformants were selected by growth on V8 medium supplemented with 50 μg/mL NPT II and screened by PCR using the primers listed in S1 Table.

### Stress sensitivity assay

To assess the sensitivity of the transformants to various stress treatments, fresh 5-mm mycelial plugs from the wild-type P6497, control (CK), and transformants were transferred from 10% V8 plates to modified V8 medium plates containing either 10 μg/mL of the alkylating agent mitomycin C (MMC) or 10 mM H_2_O_2_. The plates were incubated at 25°C in the dark for 5 days. Colonies of each strain grown on V8 plates supplemented with 0.1% DMSO served as controls (CK, depicted as DMSO in the figure). The diameter of each colony was measured, and the growth and inhibition rates were calculated. The inhibition rate was determined using the formula: (CK growth rate—Growth rate on treated plates) / CK growth rate. Each experiment was conducted in triplicate.

### Microscopic observations

*P*. *sojae* strains were cultured in clarified V8 broth at 25°C for 2 days, then washed with water to observe the localization of fluorescent fusion proteins. To assess ROS production, fungal hyphae or plant tissues were stained with 10 μM DCFH-DA (S0033S, Beyotime, Shanghai, China). To confirm the specific reaction between DCFH-DA and ROS, a control was included using 1 μM of the NADPH oxidase inhibitor diphenyleneiodonium (DPI, S8639, Selleck, Shanghai, China). DCFH-DA, with or without DPI, was directly applied to plant tissues with or without *P*. *sojae* infection. The tissues or hyphae were stained with DCFH-DA for 30 min at room temperature and visualized using a Zeiss LSM780 confocal microscope (Göttingen) at excitation/emission wavelengths of 488/525 nm. Each experiment was performed in triplicate.

### Vegetative growth and sporangium and oospore production

To determine growth rates, all strains were cultured on V8 medium at 25°C in the dark, and colony diameters were measured over 5 days. Each experiment was repeated at least three times. For sporangia production, 15 circular mycelial blocks excised from the cultures were inoculated in V8 liquid medium and incubated at 25°C in the dark for 3 days. The mycelia were then rinsed five times with sterile water at 30-minute intervals. The number of sporangia per field of view was counted under a microscope. Zoospores were counted 5 hours after sporangia formation. To detect cyst spores, 100 μL of resting spore suspension was placed on a concave glass slide and incubated in the dark at 25°C for 6 hours. Germination was defined as the presence of a germ tube longer than the diameter of the resting spore, and the germination rate was calculated. All experiments were repeated at least three times. To quantify oospore production, the strains were inoculated on V8 plates and cultured in the dark at 25°C for 9–10 days, after which the number of oospores was counted under the microscope. All experiments were repeated at least three times.

### Virulence assay

To determine the virulence of *P*. *sojae*, mycelium was inoculated into soybean hypocotyls of etiolated seedlings, and the lesion lengths were measured 48 h post-inoculation. A final concentration of 1 μM DPI was used to treat the hypocotyls of soybean seedlings in the DPI test, followed by a virulence assay. Soybean seedling tissues at 24 hours post-inoculation were incubated with PBS solution containing 5 μM H2DCFDA for 30 min at 37°C under light protection, and then ROS production was observed under a laser confocal microscope. To further evaluate differences in virulence, the relative fungal biomass in the inoculated hypocotyls at 48 hours post-inoculation was quantified by genomic DNA qPCR measurements of the ratio of pathogen to plant DNA. Amplification of the *PsACTB* from *P*. *sojae* and *GmCYP2* from *Glycine max* was performed using 50 ng of total genomic DNA from the infected material (48); the quantitative primers used are given in S1 Table. All experiments were repeated at least three times. All experiments were repeated at least three times.

### Analysis of m^6^A methylation by dot-blotting assay

To eliminate DNA contamination, total RNA from wild-type and mutant strains was treated with DNase I before extraction. The RNA was then denatured by heating at 95°C for 5 minutes. Subsequently, 300 ng of RNA was applied to a positively charged nylon membrane (GE Healthcare, USA) and dried at 37°C for 3 minutes. The membranes were further dried at 37°C for 30 minutes and UV-crosslinked using the HL-2000 system (UVP, USA) for 5 minutes. After blocking in 5% milk PBST (phosphate-buffered saline with Tween-20) for 2 hours, the membranes were incubated with an m^6^A antibody (Synaptic Systems, Wuhan, China). Following three washes with PBST at 10-minute intervals, the membranes were incubated with a secondary antibody in 5% milk PBST at room temperature for 1 hour. Visualization was performed using an enhanced chemiluminescence (ECL) detection system (Amersham Bioscience, Piscataway, USA), and relative quantification was conducted using ImageJ software.

### qRT-PCR and MeRIP-qPCR

The Quantitative reverse transcription PCR (qRT-PCR) and m6A immunoprecipitation qPCR (MeRIP-qPCR) were performed as previously described [49]. Briefly, complementary DNA (cDNA) was synthesized using the PrimeScript RT Reagent Kit with gDNA Eraser (Takara, Beijing, China) from both input RNA and immunoprecipitated RNA. Relative gene expression levels were analyzed by qRT-PCR and normalized to the input levels. The primers used for these assays are listed in S1 Table. Each experiment was conducted in triplicate. Additionally, qRT-PCR was employed to measure the intron levels of PsRCC1, serving as an indicator of transcription rate (pre-mRNA).

### m^6^A-seq and RNA-seq

Mycelia of P6497, ΔMTA1, ΔMTA2, and ΔMET16 mutants at 48 h post-inoculation (hpi) were harvested. Total RNA was isolated and purified using TRIzol reagent (Invitrogen, Carlsbad, CA, USA) according to the manufacturer’s protocol. The quantity and purity of RNA from each sample were measured with a NanoDrop ND-1000 spectrophotometer (NanoDrop, Wilmington, DE, USA). RNA integrity was assessed using a Bioanalyzer 2100 (Agilent, CA, USA), ensuring RIN values greater than 7.0, and confirmed by electrophoresis on a denaturing agarose gel. Poly(A) RNA was purified from 50 μg of total RNA using Dynabeads Oligo(dT)25-61005 (Thermo Fisher, CA, USA) through two rounds of purification. The poly(A) RNA was then fragmented using the Magnesium RNA Fragmentation Module (NEB, cat.e6150, USA) at 86°C for 7 minutes. Cleaved RNA fragments were incubated with an m6A-specific antibody (No. 202003, Synaptic Systems, Germany) in IP buffer (50 mM Tris-HCl, 750 mM NaCl, and 0.5% Igepal CA-630) for 2 hours at 4°C. The immunoprecipitated RNA was reverse-transcribed into cDNA using the SuperScript™ II Reverse Transcriptase (Invitrogen, cat. 1896649, USA). The resulting cDNA was used to synthesize U-labeled second-stranded DNA with E. coli DNA polymerase I (NEB, cat.m0209, USA), RNase H (NEB, cat.m0297, USA), and dUTP Solution (Thermo Fisher, cat.R0133, USA). An A-base was added to the blunt ends of each strand to prepare them for ligation to indexed adapters, which contain a T-base overhang for ligation to the A-tailed fragmented DNA. Single- or dual-index adapters were ligated to the fragments, followed by size selection with AMPureXP beads. The U-labeled second-stranded DNAs were treated with the heat-labile UDG enzyme (NEB, cat.m0280, USA) and then amplified by PCR under the following conditions: initial denaturation at 95°C for 3 minutes; 8 cycles of denaturation at 98°C for 15 seconds, annealing at 60°C for 15 seconds, and extension at 72°C for 30 seconds; with a final extension at 72°C for 5 minutes. The average insert size for the final cDNA library was 300±50 bp. Finally, 2×150 bp paired-end sequencing (PE150) was performed on an Illumina Novaseq™ 6000 (LC-Bio Technology Co., Ltd., Hangzhou, China) following the manufacturer’s recommended protocol.

Fastp software (https://github.com/OpenGene/fastp) was used to remove reads containing adaptor contamination, low-quality bases, and undetermined bases with default parameters. The sequence quality of both IP and input samples was also verified using Fastp. We used HISAT2 (http://daehwankimlab.github.io/hisat2) to map reads to the reference genome of *Phytophthora sojae* (Version: v). Mapped reads from the IP and input libraries were analyzed using the R package exomePeak (https://bioconductor.org/packages/exomePeak), which identifies m6A peaks in bed or bigwig format for visualization with IGV software. MEME and HOMER were employed for de novo and known motif finding, followed by motif localization relative to the peak summit. Identified peaks were annotated by intersecting with gene architecture using the R package ChIPseeker. StringTie was used to quantify expression levels for all mRNAs from input libraries by calculating FPKM (total exon fragments/mapped reads (millions) × exon length (kb)). Differentially expressed mRNAs were identified with log2(fold change) >1 or log2(fold change) < -1 and p-value < 0.05 using the R package edgeR (https://bioconductor.org/packages/edgeR).

### mRNA stability assay

The strains were cultured in V8 liquid medium for 2 days, and simultaneously, 10 μM actinomycin D and dimethylsulfoxide (DMSO) were added to the medium. After 1, 2 and 4 hours, samples were collected for downstream gene expression level detection. The primers used are listed in S1 Table. All experiments were repeated at least three times.

### γH2Ax detection

Mycelia of wild-type and mutant strains were harvested from liquid V8 cultures after 48 h incubation and washed with sterile distilled water. Proteins were isolated from vegetative hyphae as previously described^55^. Total proteins were separated by 15% SDS-PAGE gels and transferred to nitrocellulose membranes for western blot analysis. Anti-γH2Ax (ab229914, Abcam, Cambridge, UK) antibodies were used to detect the changes of histone acetylation and γH2Ax abundance in transformants. Western blotting was performed using anti-histone H4 (ab10158, Abcam, Cambridge, UK) and anti-histone H2A (ab188312, Abcam, Cambridge, UK) antibodies as loading controls. All experiments were repeated at least three times.

### Comet assay

A comet assay was conducted using a Comet Assay Kit (4250-050-K; Trevigen, Gaithersburg, MD). The comets were visualized by staining with SYBR Green I and subsequently photographed using a Zeiss LSM780 confocal microscope (Gottingen). Each experimental data point represents the mean value from three independent experiments. At least 50 nuclei were photographed for each sample, and the data were analyzed using Casp_1.2.3b1 software. All experiments were conducted independently at least three times.

### Translation efficiency assay

Translation efficiency assays were performed according to a previous report [50]. Samples were added to 8 mL of extraction buffer, shaken at 4°C for 20 minutes, and centrifuged at 16,000 g for 20 minutes at 4°C. Then, the supernatant was carefully transferred to 8 mL of sucrose buffer (1.75 M sucrose, 400 mM Tris-HCl [pH 9.0], 35 mM MgCl, 5 mM EGTA, 200 mM KCl, 5 mM DTT, 50 μg/mL chloramphenicol and 50 μg/mL cycloheximide). This removed the supernatant after centrifugation at 200,000 g for 4 hours at 4°C and then addition of 300 uL of diethyl pyrocarbonate (DEPC)-treated water to the bottom collected polysomes. Total RNA and multimeric RNA were extracted for qRT-PCR analysis. The primers used are listed in S1 Table.

## Supporting information

S1 Fig CRISPR-mediated gene knockout verification of PsMTA1, PsMTA2, and PsMET16. (**A**) Amplification validation of the complete coding region of PsMTA1. (**B**) Expression patterns of *PsMTA1*, *PsMTA2*, and *PsMET16* at different stages including mycelium (my), sporangium (sp), zoospore (zo), cystospore (cy), and at 0, 3, 6, 12, 24, 48, and 72 h post-inoculation. The asterisks indicate significant differences compared to the mycelium (my) based on Tukey’s test (**P < 0.01; ns, no significance). (**C**) CRISPR-mediated gene knockout strategy for the *PsMTA1*, *PsMTA2*, and *PsMET16*. Arrows indicate primer binding sites (see S1 Table for primer sequences). (**D**) Analysis of genomic DNA from the wild-type (P6497), control EV, Six knockout mutants, ΔMTA1-1, ΔMTA1-2, ΔMTA2-1, ΔMTA2-2, ΔMET16-1, and ΔMET16-2, and three complemented knockout mutants, ΔMTA1-C, ΔMTA2-C, and ΔMET16-C, were used for further examination using the primers shown in (**C**). (**E**) Relative transcript levels of *PsMTA1*, *PsMTA2*, and *PsMET16* in the above strains. The asterisks indicate significant differences compared to P6497 based on Tukey’s test (**P < 0.01; ns, no significance). Data in (**B** and **E**) are presented as the mean ± standard deviation from three biological replicates.
(TIF)

S2 Fig Mycelial growth of serial strains incubated for 5 days under different growth conditions. (**A**) Growth characteristics of P6497, EV, ΔMTA1-1, ΔMTA2-1, ΔMET16-1, ΔMTA1-C, ΔMTA2-C, and ΔMET16-C after 5 days on V8 medium. (**B**) Colony diameter on V8 medium. Different letters represent significant differences by one-way ANOVA (P<0.05). (**C**) Mycelial inhibition by the 10mM H_2_O_2_ relative to untreated control (DMSO) was calculated in above strains. Different letters represent significant differences by one-way ANOVA (P<0.05). (**D**) Mycelial inhibition by the 0.5 M KCl relative to DMSO was calculated in above strains. Different letters represent significant differences by one-way ANOVA (P<0.05). (**E**) Mycelial inhibition by 0.5 M sorbitol relative to DMSO was calculated in above strains. Different letters represent significant differences by one-way ANOVA (P<0.05). Data in (**B**-**E**) are presented as the mean ± standard deviation from three biological replicates.
(TIF)

S3 Fig The treatment with 1 μM DPI restored the impaired virulence of ΔMTA1-1. (**A**) Soybean seedlings were inoculated with mycelial plugs of P6497, ΔMTA1-1, ΔMTA2-1, and ΔMET16-1 and then stained at 48 hpi by DCFH-DA with or without 1 μM DPI as ROS scavenger. Disease symptoms were observed at 48 hpi of above strains when soybean seedlings were treated with H_2_O (control) or DPI. (**B**) Lesion length and (**C**) pathogen biomass (right panel) of each strain under treatments with H_2_O (control) or DPI, measured at 48 hpi. Different letters represent significant differences by one-way ANOVA (P<0.05). Data in (**B** and **C**) are presented as the mean ± standard deviation from three biological replicates.
(TIF)

S4 Fig m^6^A-seq reference genome alignment. (**A**) Cumulative distribution curve for the level of m^6^A methylation across the P6497, ΔMTA1-1, ΔMTA2-1, and ΔMET16-1. (**B**) The number of valid peaks obtained by peak calling and the number of corresponding genes. (**C**) Distribution of m^6^A peaks on gene functional elements. (**D**) The number of differential peaks and the number of corresponding transcripts in three comparison groups. (**E**) Ratio of m^6^A-modified transcripts that contained different m^6^A peak numbers among the three comparison groups. (**F**) Venn diagram analysis of hypermethylated and hypomethylated genes for ΔMTA1-1 vs P6497, ΔMTA2-1 vs P6497, and ΔMET16-1 vs P6497. (**G**) The violin plot shows the change characteristics of m6A fold enrichment in the ΔMTA1-1 vs P6497, ΔMTA2-1 vs P6497, and ΔMET16-1 vs P6497.
(TIF)

S5 Fig Validation of m^6^A-seq. Detection the m^6^A levels of six transcripts in the ΔMTA1-1 VS P6497 (**A**), ΔMTA2-1 VS P6497 (**B**), and ΔMET16-1 VS P6497 (**C**) by MeRIP-qPCR. The asterisks indicate significant differences between the WT and transformants lines based on Tukey′s test (**P < 0.01). Data in **A**-**C**) are presented as the mean ± standard deviation from three biological replicates.
(TIF)

S6 Fig Characteristics of transcriptome data enrichment. (**A**) The number of up-regulated and down-regulated transcripts in the ΔMTA1-1 VS P6497, ΔMTA2-1 VS P6497, and ΔMET16-1 VS P6497. (**B**) Heat map showing gene expression level with a statistically significant difference in the ΔMTA1-1 VS P6497, ΔMTA2-1 VS P6497, and ΔMET16-1 VS P6497 (fold change > 1.5 and P-value < 0.05).
(TIF)

S7 Fig ΔMTA1-1 mutant exhibit increased sensitivity to DNA damaging agent MMC. (**A**) Hierarchical clustering of 9 catalase genes at 48 hpi in the P6497, ΔMTA1-1, ΔMTA2-1, and ΔMET16-1. Colors represented the log_2_ (Fold difference of expression) of the genes. (**B**) Catalase activity in the mycelia of the indicated strains grown in the presence of 10 mM H_2_O_2_ or non-amended V8 medium (mock). Statistical significance of the lesion length of above strains compared to the wild-type P6497 at H_2_O treatment was determined. Different letters represent statistically significant differences according to the one-way ANOVA test (p < 0.05). The error bars indicate standard deviations (n = 3). (**C**) The degradation rate of the catalase transcripts in the P6497, ΔMTA1-1, ΔMTA2-1, and ΔMET16-1 mutants. (**D**) ΔMTA1-1 mutant exhibited increased sensitivity to DNA damaging agent MMC. Mycelial growth of the indicated strains on V8 agar medium in the presence of different DNA damaging agents (10 μg/mL MMC) with 0.1% DMSO supplement used as the control. (**E**) Mycelial growth inhibition of the indicated strains by MMC relative to DMSO was calculated as (Control−Growth rate on plates with treatment)/ Control. Data are mean ± standard deviation from three biological replicates. Statistical significance compared to the P6497 was determined using Student’s t-test (**P < 0.01, ns: not significant).
(TIF)

S8 Fig Relative transcript abundance of m6A modified transcripts derived from DNA damage response (DDR)-related genes are severely affected by PsMTA1. (**A**) Integrated Genome Viewer exhibiting the m^6^A-seq read distributions in P6497 (upper panel) and ΔMTA1-1 (lower panel). Red reads originate from m6A IP libraries of WT and blue reads originate from m6A IP libraries of the KO. (**B**) Detection of the m^6^A modification levels in P6497 and ΔMTA1-1 by MeRIP-qPCR. Statistical significance compared to the P6497 was determined using Student’s t-test (**P < 0.01). (**C**) Strand-specific qRT-PCR analysis. Total mRNA was used to perform reverse transcription using specific primer priming the intron region of DDR genes (intron of actin gene was used as a control). qRT-PCR was then performed to amplify the DDR genes intron to demonstrate the transcription rate of DDR genes. Statistical significance of above strains compared to the P6497 was determined using Student’s t-test (ns, no significance). (**D**) Relative expression of DDR genes was detected by qRT-PCR in P6497 and ΔMTA1-1. Statistical significance compared to the P6497 was determined using Student’s t-test (**P < 0.01). Data in (**B**-**D**) are presented as the mean ± standard deviation from three biological replicates.
(TIF)

S9 Fig CRISPR-mediated gene editing verification of *PsRCC1*. (**A**) *Phytophthora sojae* RCC1 protein conserved domains predicted using a simple modular architecture research tool (Smart). (**B**) Analysis of genomic DNA from the wild-type (P6497), control EV, ΔRCC1-1, ΔRCC1-2, ΔRCC1/RCC1, and ΔRCC1/RCC1^A2961C^, were used for further examination using the primers shown in S1 Table. (**C**) Relative transcript levels of *PsRCC1* in the above strains. The asterisks indicate significant differences compared to P6497 based on Tukey’s test (**P < 0.01; ns, no significance). (**D**) Translation efficiency of the mRNA in the WT and transformants. Total RNA and polysome RNA were extracted from mycelia cultivated in V8 liquid medium. The abundance ratio of transcripts, i.e., the polysomal RNA against the total RNA, indicated the translation efficiency. The asterisks indicate significant differences based on Tukey’s test (ns, no significance). The error bars indicate standard deviations (n = 3). (**E**) Expression patterns of *PsRCC1* at different stages including mycelium (my), sporangium (sp), zoospore (zo), cystospore (cy), and at 0, 3, 6, 12, 24, 48, and 72 h post-inoculation. The asterisks indicate significant differences compared to the mycelium (my) based on Tukey’s test (**P < 0.01; ns, no significance). Data in (**C**-**E**) are presented as the mean ± standard deviation from three biological replicates.
(TIF)

S10 Fig Five DDR genes, *PsMyb*, *PsCCA1*, *PsGNAT*, *PsBDF1*, and *PsZFAND3*, were not involved in the H_2_O_2_ stress response. (**A**) Analysis of genomic DNA from the wild-type (P6497), DDR genes-knockout (ΔCCA1, ΔGNAT, and ΔZFAND3) using the primers shown in S1C Fig. (**B**) Schematic diagram of gene silencing. (**C**) Expression analyses of *PsMyb*, *PsCCA1*, *PsGNAT*, *PsBDF1*, *and PsZFAND3* by reverse transcription-polymerase chain reaction (RT-PCR) in above lines. Data are presented as the mean ± standard deviation from three biological replicates. Statistical significance compared to the P6497 was determined using Student’s t-test (**P < 0.01). (**D**) DDR mutants exhibited no significantly different sensitivity to DNA damaging agents. Left panels: Mycelial growth of the indicated strains on V8 agar medium in the presence of 10 mM H_2_O, with 0,01% DMSO used as the control. Right panels: growth inhibition was calculated as (Control−Growth rate on plates with treatment)/ Control. Statistical significance compared to the P6497 was determined using Student’s t-test (ns: not significant). Data in (**C** and **D**) are presented as the mean ± standard deviation from three biological replicates.
(TIF)

S11 Fig Overexpression strain of PsRCC1 in ΔMTA1 mutant verification. (**A**) Schematic diagram of *PsRCC1* gene overexpression. (**B**) Relative transcript levels of *PsRCC1* in the P6497 and ΔMTA1/RCC1. The asterisks indicate significant differences compared to P6497 based on Tukey’s test (**P < 0.01). Data are presented as the mean ± standard deviation from three biological replicates. (**C**) Expression of PsRCC1 protein was detected by western blotting with anti-FLAG.
(TIF)

S12 Fig PsMTA1 regulate ROS-induced γH2Ax enrichment in an m^6^A-dependent manner. (**A**) The elevation of γH2Ax levels in response to 10mM H_2_O_2_ was inhibited in ΔRCC1-1 and ΔRCC1-2 mutants compared to that in P6497, ΔRCC1/RCC1, and ΔMTA1/RCC1. γH2Ax levels were determined by western blotting using anti-γH2Ax antibody. Detection of H2A protein was used as the loading control. (**B**) The intensity of the γH2Ax band from P6497 treated with DMSO was set as 1.00; and the relative intensity of γH2Ax band from each treatment was quantified with Image J. Different letters represent significant differences by one-way ANOVA (P < 0.05). (**C**) The elevation of γH2Ax levels in response to 10 μg/mL MMC was inhibited in ΔRCC1-1 and ΔRCC1-2 mutants compared to that in P6497, ΔRCC1/RCC1, and ΔMTA1/RCC1. γH2Ax levels were determined by western blotting using anti-γH2Ax antibody. Detection of H2A protein was used as the loading control. (**D**) The intensity of the γH2Ax band from P6497 treated with DMSO was set as 1.00; and the relative intensity of γH2Ax band from each treatment was quantified with Image J. Different letters represent significant differences by one-way ANOVA (P < 0.05). Data in (**B** and **D**) are presented as the mean ± standard deviation from three biological replicates.
(TIF)

S1 Table Primers used in this study. (XLSX)

S2 Table A summary of m6A-seq results in P6497, ΔMTA1-1, ΔMTA2-1, and ΔMET16-1 mutants. (XLSX)

S3 Table Different peaks in ΔMTA1-1, ΔMTA2-1, and ΔMET16-1 mutants compared to P6497. (XLSX)

S4 Table Differentially expressed genes in ΔMTA1-1, ΔMTA2-1, and ΔMET16-1 mutants compared to P6497. (XLSX)

S5 Table DNA damage response genes. (XLSX)

S6 Table Phenotypes of ΔRCC1 and ΔRCC1/RCC1^A2961C^. (XLSX)

S1 Data The underlying numerical data and statistical analysis. (XLSX)
