# Supplementary figures and images for: The novel roles of RNA m6A modification in regulating the development, infection, and oxidative DNA damage repair of Phytophthora sojae
Source: PLoS Pathog. 2024 Sep 23;20(9):e1012553. doi: 10.1371/journal.ppat.1012553 (PMC11449341; doi:10.1371/journal.ppat.1012553)

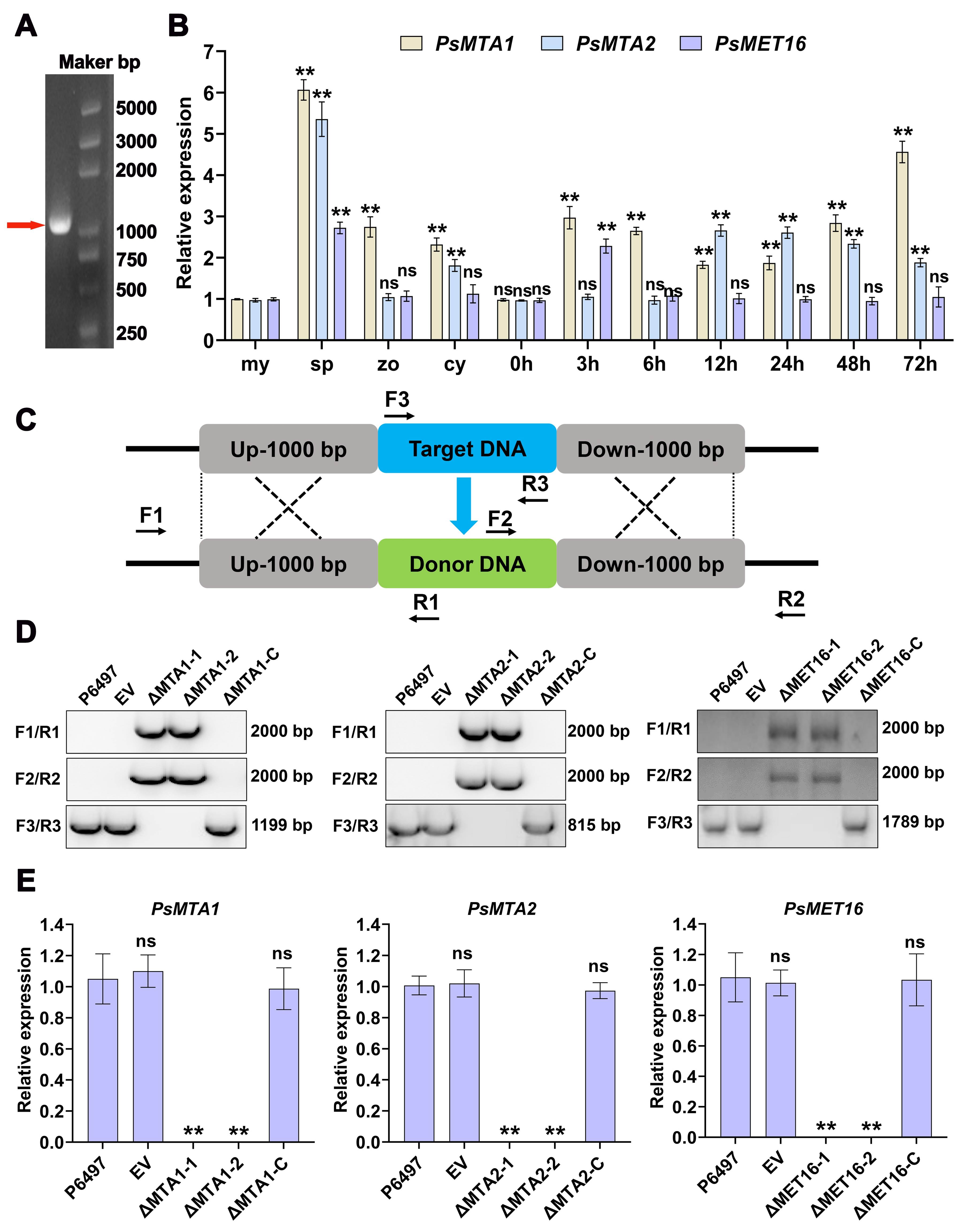

Supplement: S1 Fig — (A) Amplification validation of the complete coding region of PsMTA1. (B) Expression patterns of PsMTA1, PsMTA2, and PsMET16 at different stages including mycelium (my), sporangium (sp), zoospore (zo), cystospore (cy), and at 0, 3, 6, 12, 24, 48, and 72 h post-inoculation. The asterisks indicate significant differences compared to the mycelium (my) based on Tukey’s test (**P < 0.01; ns, no significance). (C) CRISPR-mediated gene knockout strategy for the PsMTA1, PsMTA2, and PsMET16. Arrows indicate primer binding sites (see S1 Table for primer sequences). (D) Analysis of genomic DNA from the wild-type (P6497), control EV, Six knockout mutants, ΔMTA1-1, ΔMTA1-2, ΔMTA2-1, ΔMTA2-2, ΔMET16-1, and ΔMET16-2, and three complemented knockout mutants, ΔMTA1-C, ΔMTA2-C, and ΔMET16-C, were used for further examination using the primers shown in (C). (E) Relative transcript levels of PsMTA1, PsMTA2, and PsMET16 in the above strains. The asterisks indicate significant differences compared to P6497 based on Tukey’s test (**P < 0.01; ns, no significance). Data in (B and E) are presented as the mean ± standard deviation from three biological replicates. (TIF) [file ppat.1012553.s001.tif]

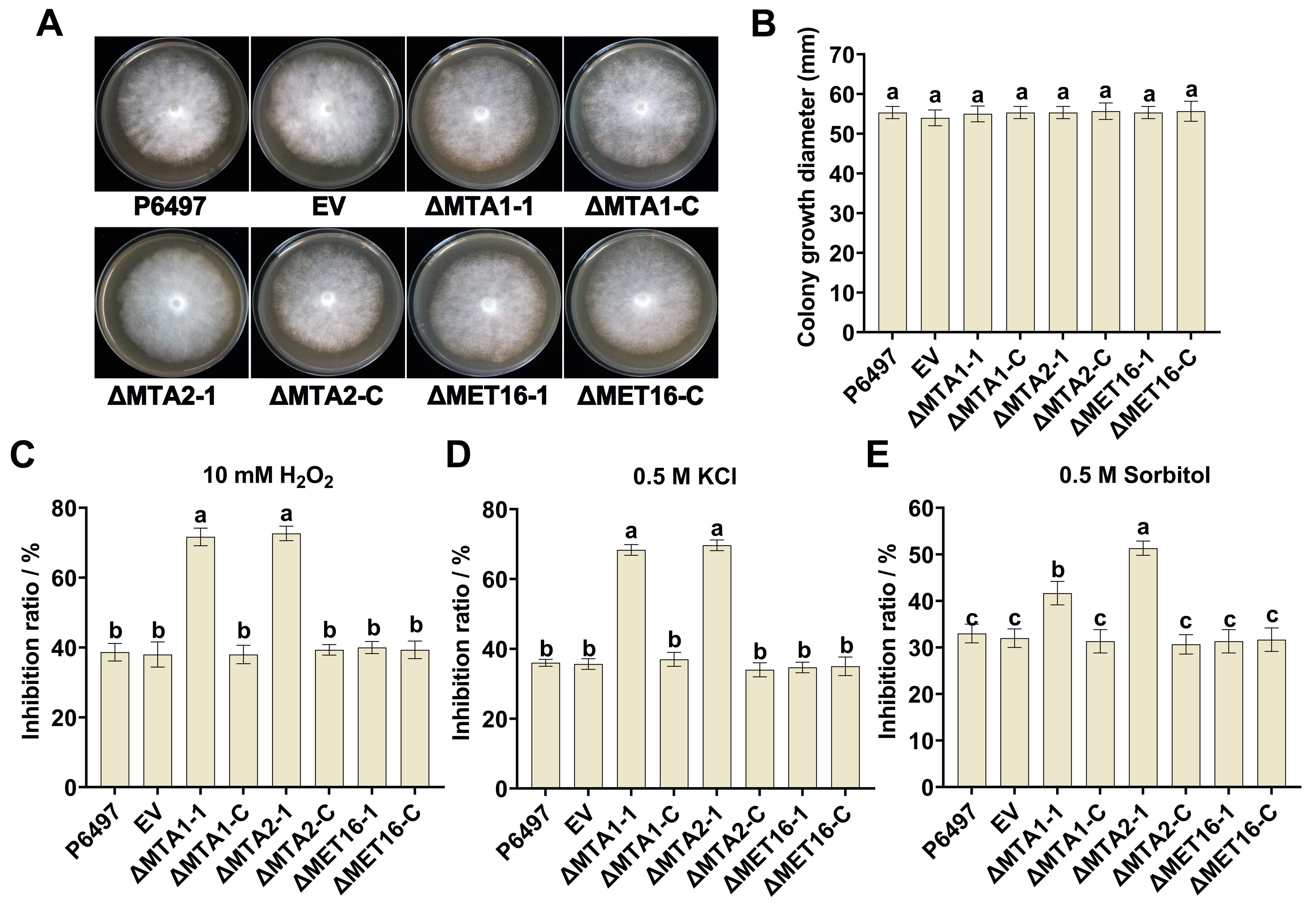

Supplement: S2 Fig — (A) Growth characteristics of P6497, EV, ΔMTA1-1, ΔMTA2-1, ΔMET16-1, ΔMTA1-C, ΔMTA2-C, and ΔMET16-C after 5 days on V8 medium. (B) Colony diameter on V8 medium. Different letters represent significant differences by one-way ANOVA (P<0.05). (C) Mycelial inhibition by the 10mM H2O2 relative to untreated control (DMSO) was calculated in above strains. Different letters represent significant differences by one-way ANOVA (P<0.05). (D) Mycelial inhibition by the 0.5 M KCl relative to DMSO was calculated in above strains. Different letters represent significant differences by one-way ANOVA (P<0.05). (E) Mycelial inhibition by 0.5 M sorbitol relative to DMSO was calculated in above strains. Different letters represent significant differences by one-way ANOVA (P<0.05). Data in (B-E) are presented as the mean ± standard deviation from three biological replicates. (TIF) [file ppat.1012553.s002.tif]

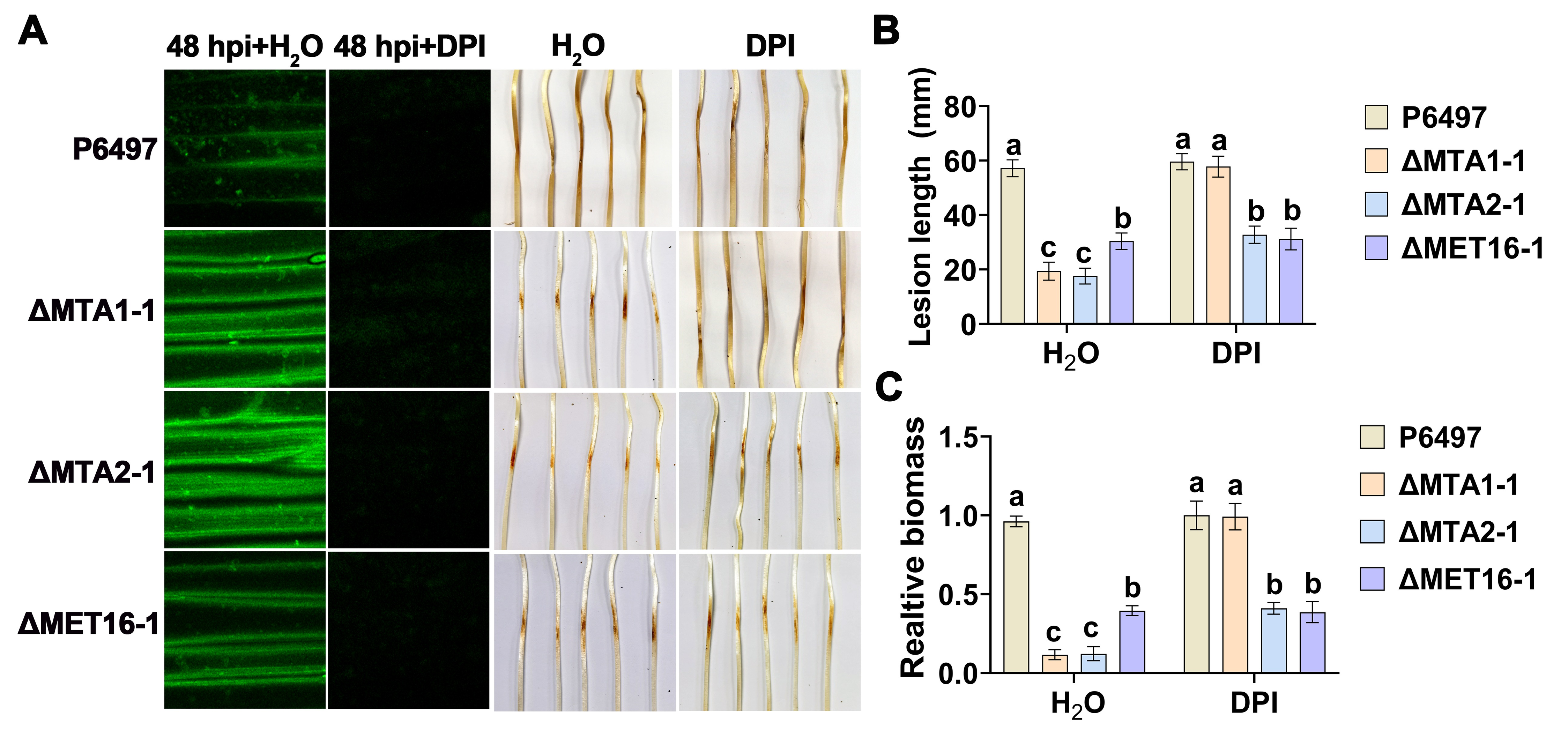

Supplement: S3 Fig — (A) Soybean seedlings were inoculated with mycelial plugs of P6497, ΔMTA1-1, ΔMTA2-1, and ΔMET16-1 and then stained at 48 hpi by DCFH-DA with or without 1 μM DPI as ROS scavenger. Disease symptoms were observed at 48 hpi of above strains when soybean seedlings were treated with H2O (control) or DPI. (B) Lesion length and (C) pathogen biomass (right panel) of each strain under treatments with H2O (control) or DPI, measured at 48 hpi. Different letters represent significant differences by one-way ANOVA (P<0.05). Data in (B and C) are presented as the mean ± standard deviation from three biological replicates. (TIF) [file ppat.1012553.s003.tif]

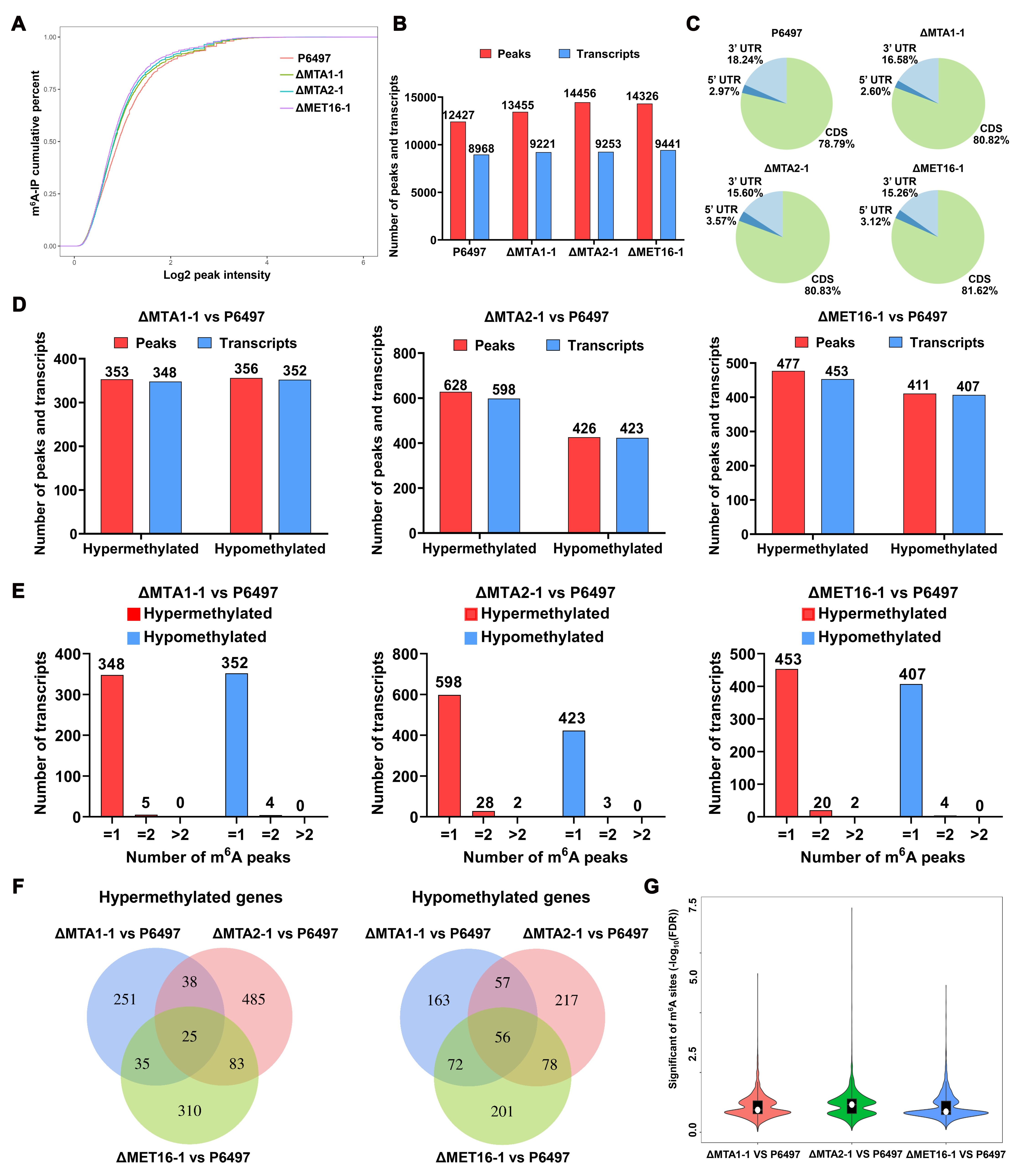

Supplement: S4 Fig — (A) Cumulative distribution curve for the level of m6A methylation across the P6497, ΔMTA1-1, ΔMTA2-1, and ΔMET16-1. (B) The number of valid peaks obtained by peak calling and the number of corresponding genes. (C) Distribution of m6A peaks on gene functional elements. (D) The number of differential peaks and the number of corresponding transcripts in three comparison groups. (E) Ratio of m6A-modified transcripts that contained different m6A peak numbers among the three comparison groups. (F) Venn diagram analysis of hypermethylated and hypomethylated genes for ΔMTA1-1 vs P6497, ΔMTA2-1 vs P6497, and ΔMET16-1 vs P6497. (G) The violin plot shows the change characteristics of m6A fold enrichment in the ΔMTA1-1 vs P6497, ΔMTA2-1 vs P6497, and ΔMET16-1 vs P6497. (TIF) [file ppat.1012553.s004.tif]

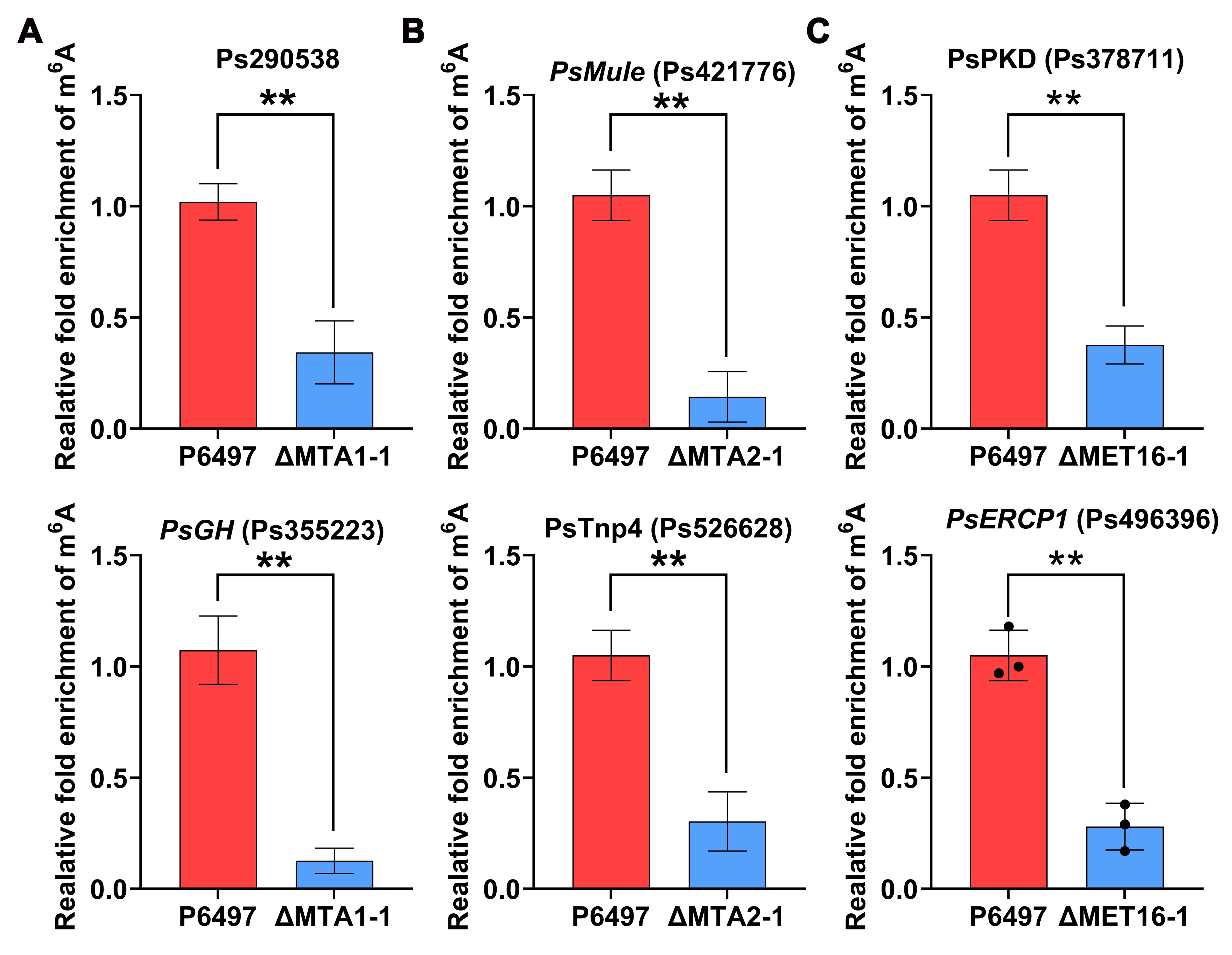

Supplement: S5 Fig — Detection the m6A levels of six transcripts in the ΔMTA1-1 VS P6497 (A), ΔMTA2-1 VS P6497 (B), and ΔMET16-1 VS P6497 (C) by MeRIP-qPCR. The asterisks indicate significant differences between the WT and transformants lines based on Tukey′s test (**P < 0.01). Data in A-C) are presented as the mean ± standard deviation from three biological replicates. (TIF) [file ppat.1012553.s005.tif]

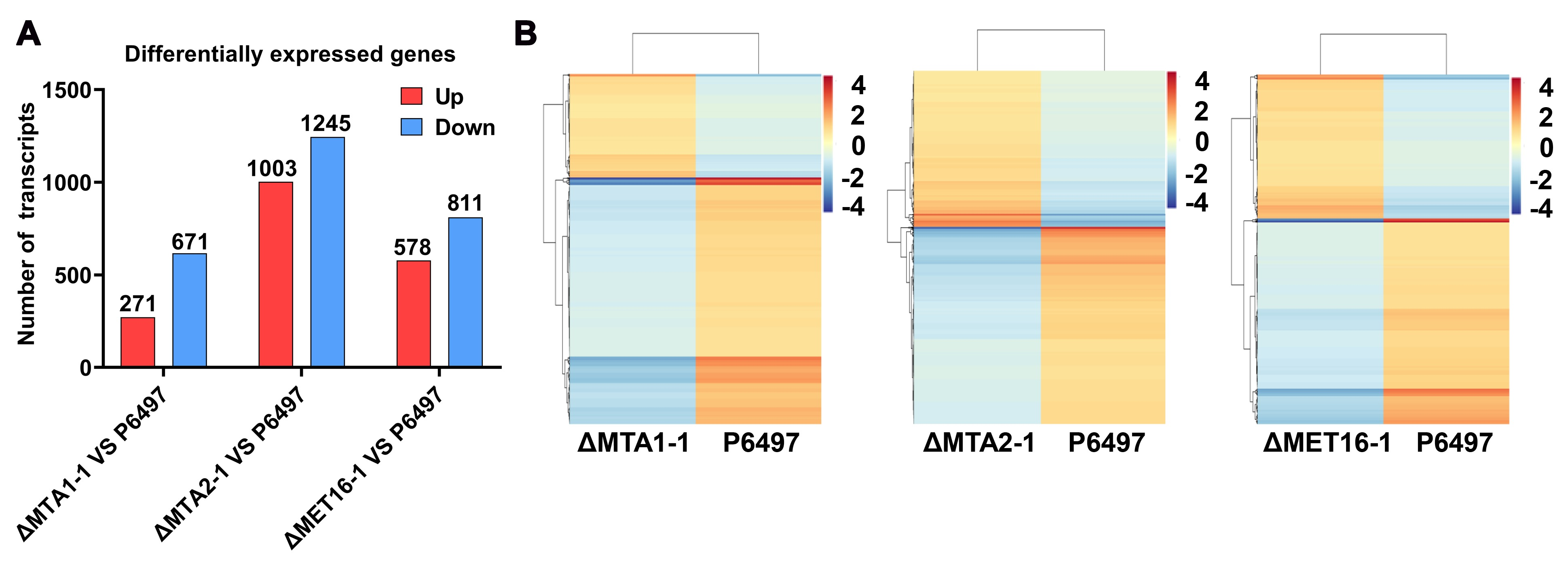

Supplement: S6 Fig — (A) The number of up-regulated and down-regulated transcripts in the ΔMTA1-1 VS P6497, ΔMTA2-1 VS P6497, and ΔMET16-1 VS P6497. (B) Heat map showing gene expression level with a statistically significant difference in the ΔMTA1-1 VS P6497, ΔMTA2-1 VS P6497, and ΔMET16-1 VS P6497 (fold change > 1.5 and P-value < 0.05). (TIF) [file ppat.1012553.s006.tif]

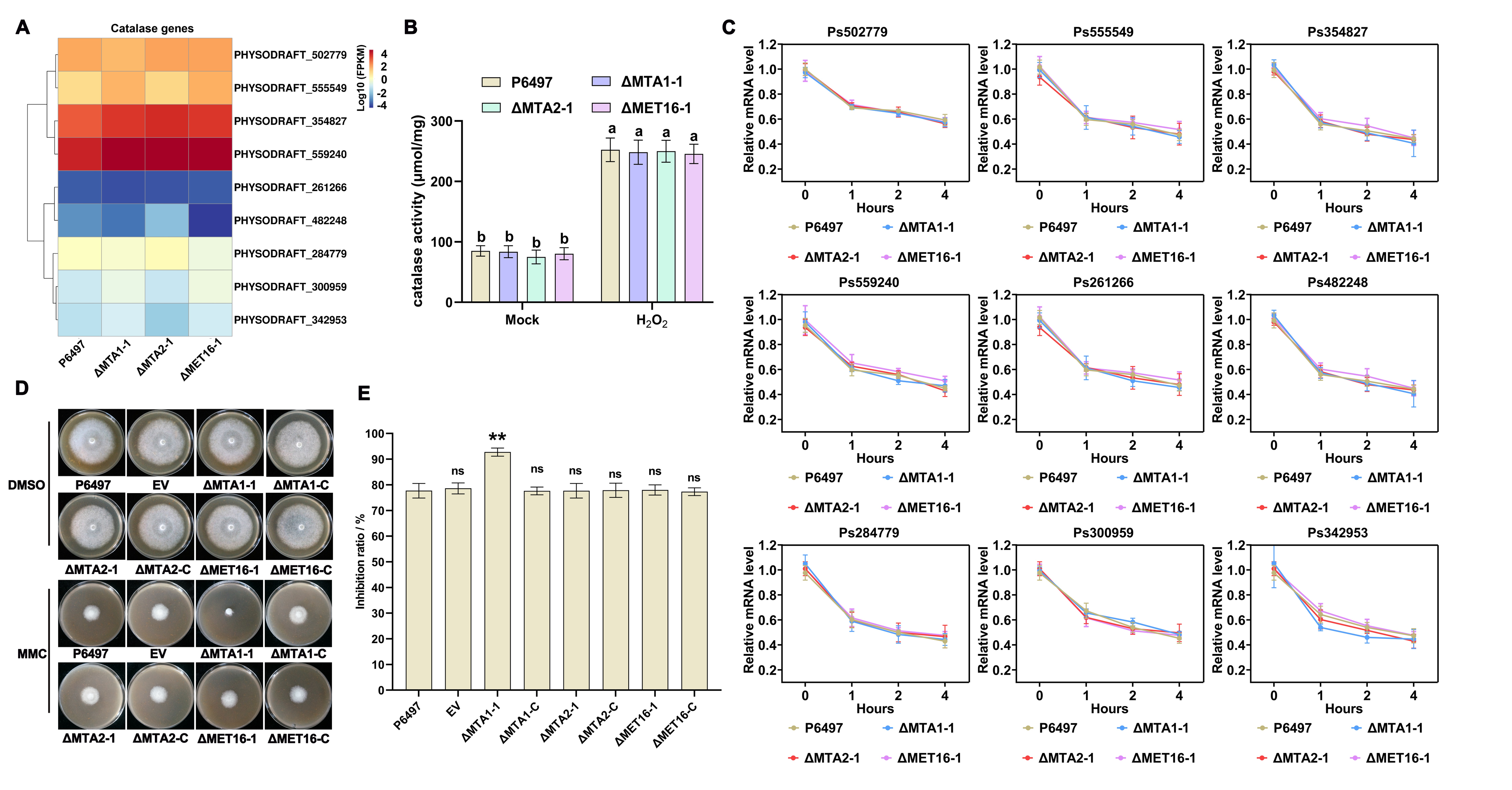

Supplement: S7 Fig — (A) Hierarchical clustering of 9 catalase genes at 48 hpi in the P6497, ΔMTA1-1, ΔMTA2-1, and ΔMET16-1. Colors represented the log2 (Fold difference of expression) of the genes. (B) Catalase activity in the mycelia of the indicated strains grown in the presence of 10 mM H2O2 or non-amended V8 medium (mock). Statistical significance of the lesion length of above strains compared to the wild-type P6497 at H2O treatment was determined. Different letters represent statistically significant differences according to the one-way ANOVA test (p < 0.05). The error bars indicate standard deviations (n = 3). (C) The degradation rate of the catalase transcripts in the P6497, ΔMTA1-1, ΔMTA2-1, and ΔMET16-1 mutants. (D) ΔMTA1-1 mutant exhibited increased sensitivity to DNA damaging agent MMC. Mycelial growth of the indicated strains on V8 agar medium in the presence of different DNA damaging agents (10 μg/mL MMC) with 0.1% DMSO supplement used as the control. (E) Mycelial growth inhibition of the indicated strains by MMC relative to DMSO was calculated as (Control−Growth rate on plates with treatment)/ Control. Data are mean ± standard deviation from three biological replicates. Statistical significance compared to the P6497 was determined using Student’s t-test (**P < 0.01, ns: not significant). (TIF) [file ppat.1012553.s007.tif]

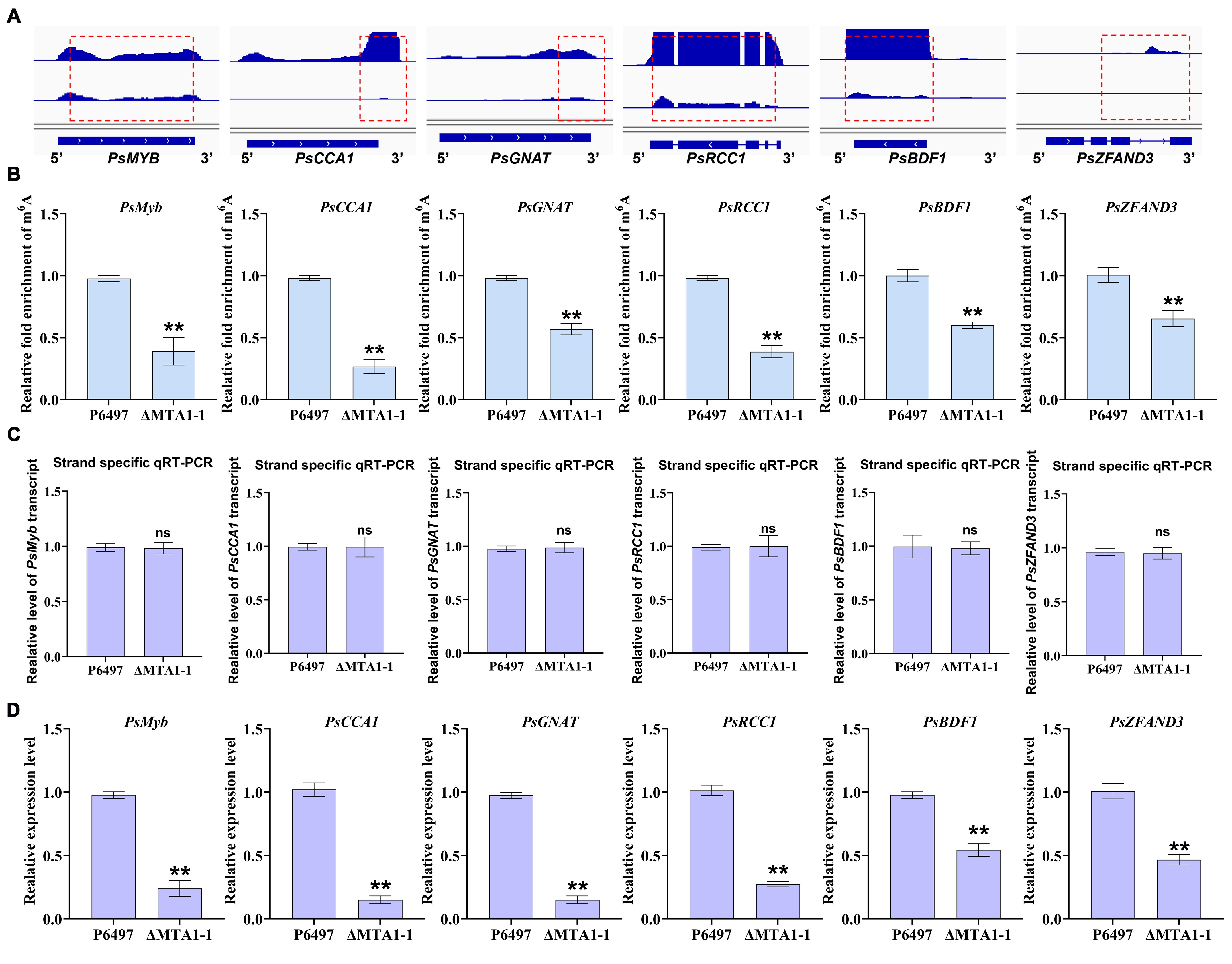

Supplement: S8 Fig — (A) Integrated Genome Viewer exhibiting the m6A-seq read distributions in P6497 (upper panel) and ΔMTA1-1 (lower panel). Red reads originate from m6A IP libraries of WT and blue reads originate from m6A IP libraries of the KO. (B) Detection of the m6A modification levels in P6497 and ΔMTA1-1 by MeRIP-qPCR. Statistical significance compared to the P6497 was determined using Student’s t-test (**P < 0.01). (C) Strand-specific qRT-PCR analysis. Total mRNA was used to perform reverse transcription using specific primer priming the intron region of DDR genes (intron of actin gene was used as a control). qRT-PCR was then performed to amplify the DDR genes intron to demonstrate the transcription rate of DDR genes. Statistical significance of above strains compared to the P6497 was determined using Student’s t-test (ns, no significance). (D) Relative expression of DDR genes was detected by qRT-PCR in P6497 and ΔMTA1-1. Statistical significance compared to the P6497 was determined using Student’s t-test (**P < 0.01). Data in (B-D) are presented as the mean ± standard deviation from three biological replicates. (TIF) [file ppat.1012553.s008.tif]

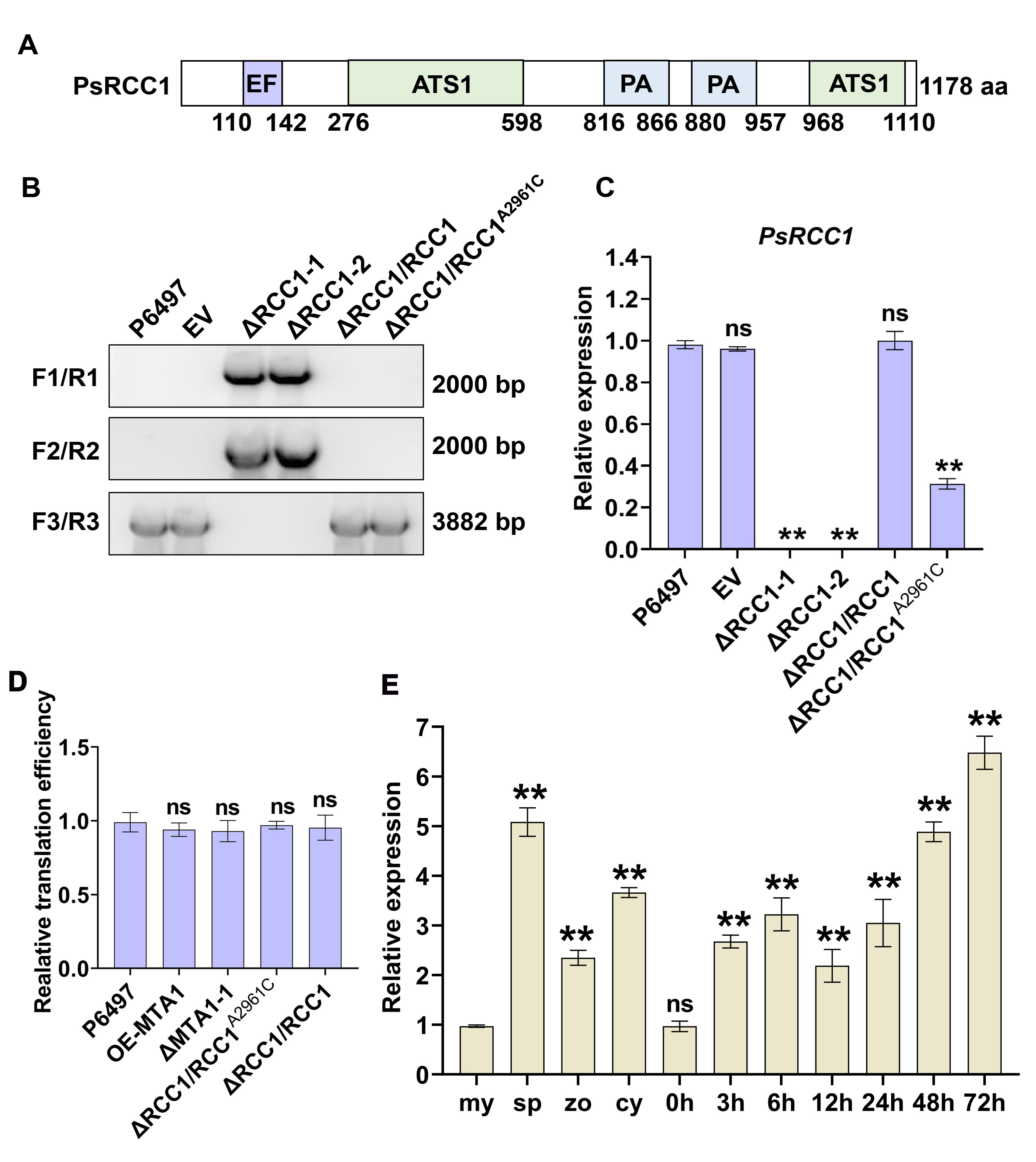

Supplement: S9 Fig — (A) Phytophthora sojae RCC1 protein conserved domains predicted using a simple modular architecture research tool (Smart). (B) Analysis of genomic DNA from the wild-type (P6497), control EV, ΔRCC1-1, ΔRCC1-2, ΔRCC1/RCC1, and ΔRCC1/RCC1A2961C, were used for further examination using the primers shown in S1 Table. (C) Relative transcript levels of PsRCC1 in the above strains. The asterisks indicate significant differences compared to P6497 based on Tukey’s test (**P < 0.01; ns, no significance). (D) Translation efficiency of the mRNA in the WT and transformants. Total RNA and polysome RNA were extracted from mycelia cultivated in V8 liquid medium. The abundance ratio of transcripts, i.e., the polysomal RNA against the total RNA, indicated the translation efficiency. The asterisks indicate significant differences based on Tukey’s test (ns, no significance). The error bars indicate standard deviations (n = 3). (E) Expression patterns of PsRCC1 at different stages including mycelium (my), sporangium (sp), zoospore (zo), cystospore (cy), and at 0, 3, 6, 12, 24, 48, and 72 h post-inoculation. The asterisks indicate significant differences compared to the mycelium (my) based on Tukey’s test (**P < 0.01; ns, no significance). Data in (C-E) are presented as the mean ± standard deviation from three biological replicates. (TIF) [file ppat.1012553.s009.tif]

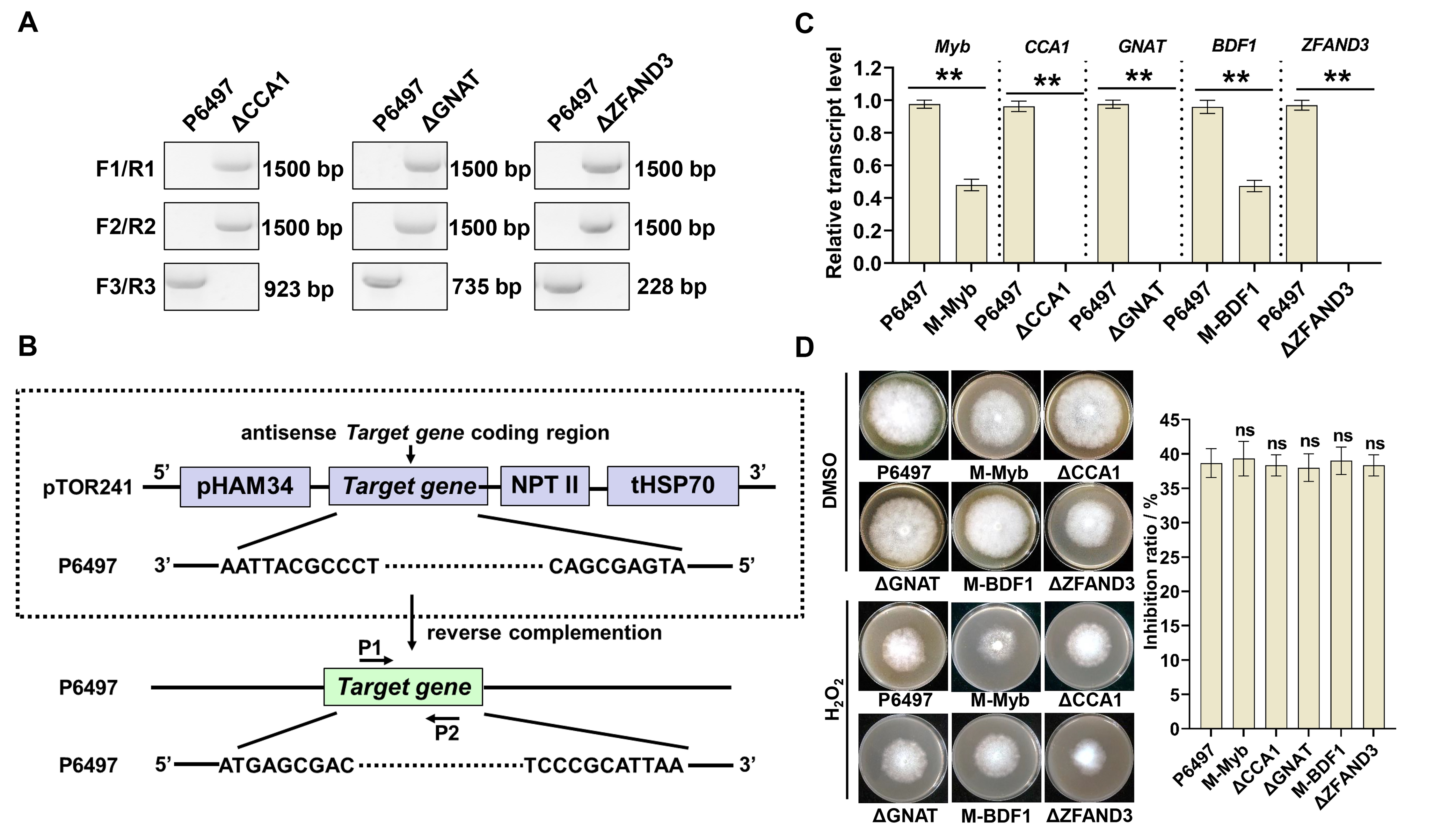

Supplement: S10 Fig — (A) Analysis of genomic DNA from the wild-type (P6497), DDR genes-knockout (ΔCCA1, ΔGNAT, and ΔZFAND3) using the primers shown in S1C Fig. (B) Schematic diagram of gene silencing. (C) Expression analyses of PsMyb, PsCCA1, PsGNAT, PsBDF1, and PsZFAND3 by reverse transcription-polymerase chain reaction (RT-PCR) in above lines. Data are presented as the mean ± standard deviation from three biological replicates. Statistical significance compared to the P6497 was determined using Student’s t-test (**P < 0.01). (D) DDR mutants exhibited no significantly different sensitivity to DNA damaging agents. Left panels: Mycelial growth of the indicated strains on V8 agar medium in the presence of 10 mM H2O, with 0,01% DMSO used as the control. Right panels: growth inhibition was calculated as (Control−Growth rate on plates with treatment)/ Control. Statistical significance compared to the P6497 was determined using Student’s t-test (ns: not significant). Data in (C and D) are presented as the mean ± standard deviation from three biological replicates. (TIF) [file ppat.1012553.s010.tif]

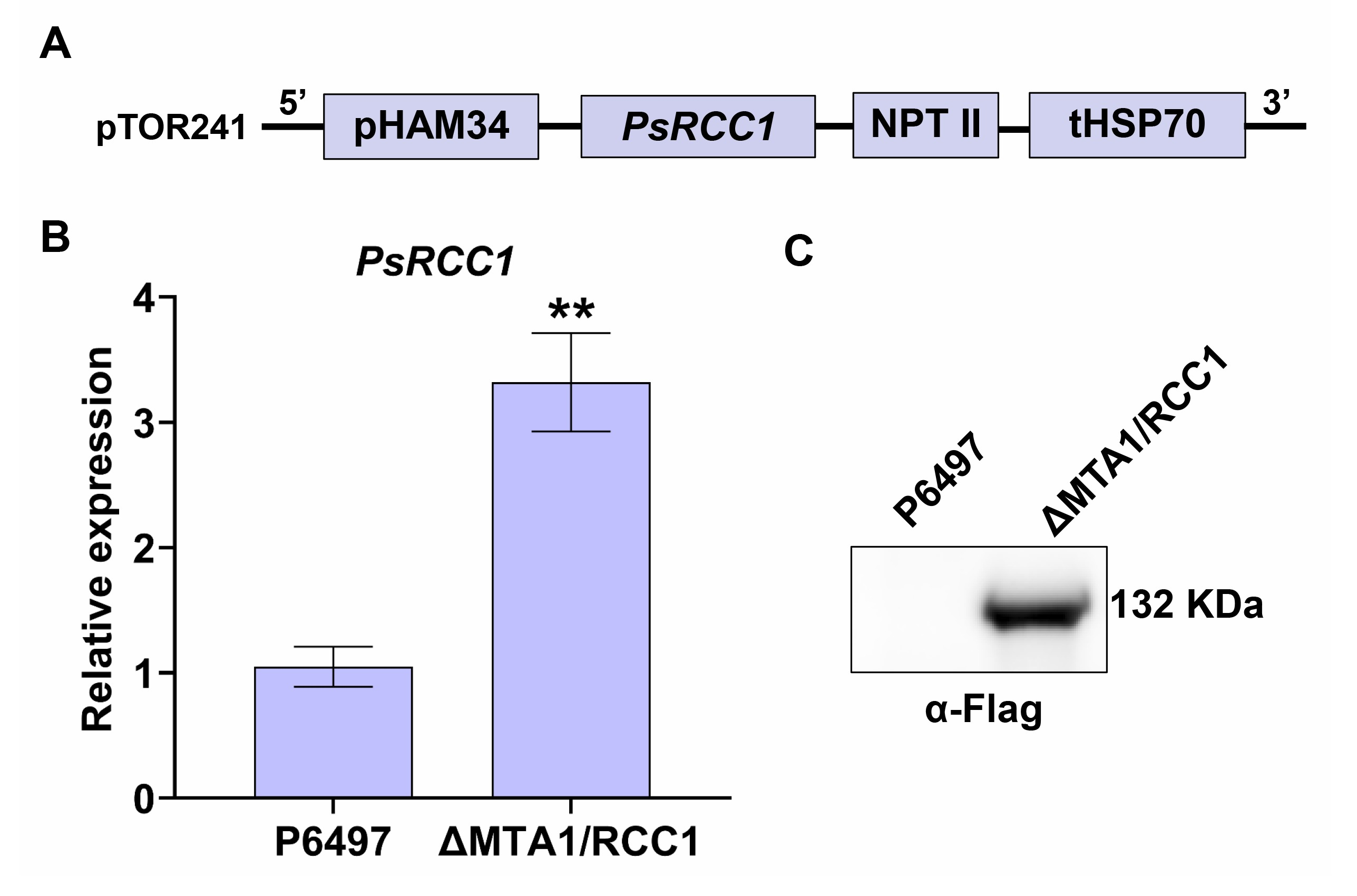

Supplement: S11 Fig — (A) Schematic diagram of PsRCC1 gene overexpression. (B) Relative transcript levels of PsRCC1 in the P6497 and ΔMTA1/RCC1. The asterisks indicate significant differences compared to P6497 based on Tukey’s test (**P < 0.01). Data are presented as the mean ± standard deviation from three biological replicates. (C) Expression of PsRCC1 protein was detected by western blotting with anti-FLAG. (TIF) [file ppat.1012553.s011.tif]

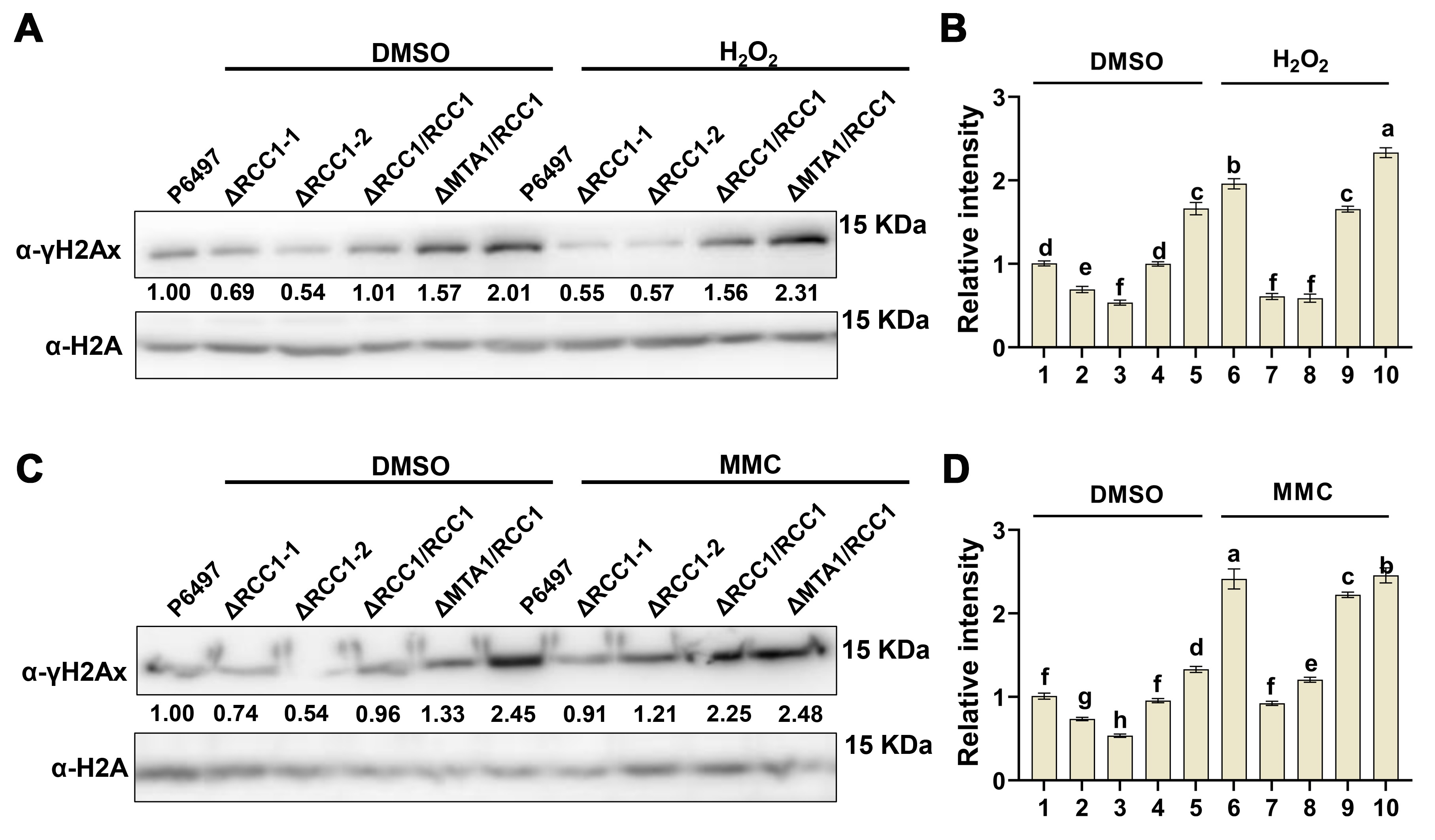

Supplement: S12 Fig — (A) The elevation of γH2Ax levels in response to 10mM H2O2 was inhibited in ΔRCC1-1 and ΔRCC1-2 mutants compared to that in P6497, ΔRCC1/RCC1, and ΔMTA1/RCC1. γH2Ax levels were determined by western blotting using anti-γH2Ax antibody. Detection of H2A protein was used as the loading control. (B) The intensity of the γH2Ax band from P6497 treated with DMSO was set as 1.00; and the relative intensity of γH2Ax band from each treatment was quantified with Image J. Different letters represent significant differences by one-way ANOVA (P < 0.05). (C) The elevation of γH2Ax levels in response to 10 μg/mL MMC was inhibited in ΔRCC1-1 and ΔRCC1-2 mutants compared to that in P6497, ΔRCC1/RCC1, and ΔMTA1/RCC1. γH2Ax levels were determined by western blotting using anti-γH2Ax antibody. Detection of H2A protein was used as the loading control. (D) The intensity of the γH2Ax band from P6497 treated with DMSO was set as 1.00; and the relative intensity of γH2Ax band from each treatment was quantified with Image J. Different letters represent significant differences by one-way ANOVA (P < 0.05). Data in (B and D) are presented as the mean ± standard deviation from three biological replicates. (TIF) [file ppat.1012553.s012.tif]
